# Xylylethynyl Titanocene with a Microsecond Emission Lifetime Photosensitizes Singlet-Oxygen Formation and Photon Upconversion

**DOI:** 10.1021/acs.inorgchem.5c01773

**Published:** 2025-07-17

**Authors:** Jack M. Sledesky, John H. Zimmerman, Henry C. London, Ethan C. Lambert, Colin D. McMillen, Matilda Barker, Kenneth Hanson, Paul S. Wagenknecht

**Affiliations:** † Department of Chemistry, 3628 Furman University, Greenville, South Carolina 29609, United States; ‡ Department of Chemistry and Biochemistry, 7823 Florida State University, Tallahassee, Florida 32306, United States; § Department of Chemistry, 2545 Clemson University, Clemson, South Carolina 29634, United States

## Abstract

Coordination complexes containing d^0^ metals with long-lived ligand-to-metal charge transfer (LMCT) excited states are promising candidates for use as photosensitizers. Previously, Cp*_2_Ti­(C_2_Ph)_2_CuBr (where Cp* = pentamethylcyclopentadienyl and C_2_Ph = phenylethynyl) was reported to be emissive in THF solution at room temperature (RT) from an excited state with significant Cp*-to-Ti LMCT character (λ_em_ = 693 nm, τ = 0.18 μs). However, the corresponding cyclopentadienyl complex was not emissive. Structural constraint was hypothesized as a reason for the enhanced photophysics of the Cp* complex. To further test this hypothesis, the corresponding complex with *o*-xylylethynyl ligands, Cp*_2_Ti­(C_2_PhMe_2_)_2_CuBr, has been prepared and characterized. X-ray crystallography demonstrates significant steric congestion caused by the additional methyl substituents. This xylylethynyl complex is emissive in THF solution at RT and the lifetime is approximately 10-fold greater (λ_em_ = 734 nm, τ = 1.6 μs) than the corresponding phenylethynyl complex. Spectroscopic and computational data are consistent with this emission being phosphorescence with significant Cp*-to-Ti and xylylethynyl-to-Ti LMCT character. The evidence is also consistent with an excited state that is less distorted for the xylylethynyl complex than the phenylethynyl complex. The sterically induced, long lifetime of the xylylethynyl complex enabled its use in photon upconversion and ^1^O_2_ formation.

## Graphical abstract

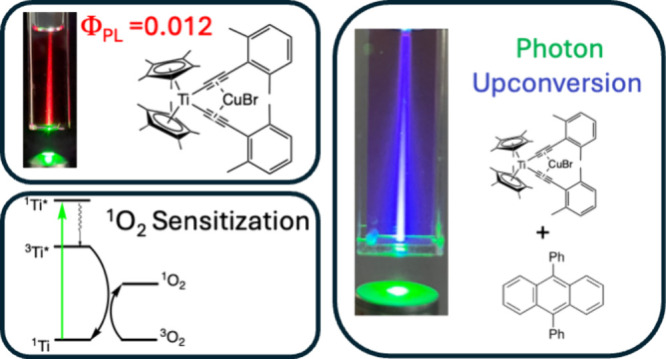

## Introduction

Over the past decade, there has been a significant effort toward developing photocatalysts of earth-abundant transition metals to augment or replace the well-known systems based on metal-to-ligand charge transfer (MLCT) excited states in ruthenium and iridium complexes.^1–3^ Much of this research has focused on first-row transition metals but these typically suffer from short excited-state lifetimes due to thermally accessible, short-lived, metal-centered (MC) excited states.^4^ Much effort has been expended to increase the energy of these MC states through carefully designed high-field ligands and this has resulted in extended lifetimes for complexes of chromium(0),^5,6^ manganese­(I),^5–7^ iron­(II),^8–10^ iron­(III),^11–14^ cobalt­(III),^15,16^ and nickel­(II).^17^ Another strategy has been to take advantage of the MC, intraconfigurational, doublet excited states of d^3^ systems such as chromium­(III) and vanadium­(II), where the doublet excited-state and ground-state potential wells are nested.^18–20^ These nested potential wells result in slow nonradiative decay, and in such cases, the MC state is typically the photoactive state, though a related d^3^ complex of manganese­(IV) shows photoreactivity stemming from a state of ^2^LMCT character.^21^ Yet another strategy involves the design of systems devoid of MC excited states altogether. For example, the d^10^ configuration of copper­(I) eliminates the MC states, and many such systems with MLCT excited states have been developed with lifetimes on the order of microseconds in room-temperature (RT) fluid solution.^22–27^

Recently, this strategy of eliminating MC states has focused on complexes of d^0^ metals.^28^ The high-valent metals typically employed are often stabilized by electron-rich ligands, resulting in a reversal of the direction of the electron flow in the lowest-energy electronic transition (relative to MLCT transitions). Thus, such complexes are dominated by ligand-to-metal charge-transfer (LMCT) excited states. In general, there has been recent interest in photoactive LMCT states,^29–31^ particularly with d^5^ metals.^11–14,32^ However, the d^0^ metals have the advantage of eliminating the often deleterious MC states. A notable example of such a strategy involves exploiting the LMCT excited state of pyridine dipyrrolide complexes of Zr^IV^.^33–35^ Such complexes have lifetimes on the order of hundreds of microseconds and have been successfully used to sensitize photoreactions and molecular photon upconversion. Recently, several Zr^IV^ and Hf^IV^ complexes with substituted cyclopentadienyl ligands have also been developed with long-lived LMCT excited states and shown to efficiently photosensitize singlet-oxygen formation.^36–38^ Prior to these reports, several d^0^ complexes with emissive LMCT excited states with lifetimes on the order of microseconds in RT fluid solution had been reported, with Group 3, 4, and 5 metallocenes being prominent.^39–46^ It is also noteworthy that Cp_2_TiCl_2_ has recently been demonstrated to be a photocatalyst, despite a lack of emission in RT fluid solution.^47,48^

While several Zr^IV^ complexes are emissive from an LMCT state in RT fluid solution, the corresponding Ti^IV^ systems are not.^33,37,49^ Thus, we have recently been interested in developing emissive Ti^IV^ complexes with excited-state lifetimes sufficient to engage in collisional reactivity.^50–53^ In 2022, we reported the first room-temperature emission from a titanium­(IV) complex in solution (Φ_PL_ ≈ 2 × 10^–4^), which was achieved using Cp_2_Ti­(C_2_Ph)_2_ (^**Ph**^[Ti] in Figure 1).^52^ However, ^**Ph**^[Ti] undergoes photodecomposition with a quantum yield, Φ_decomp_, of 0.65 in dearated THF. The coordination of CuBr between the alkynes (^**Ph**^ **[Ti]­CuBr** in Figure 1), lowers Φ_decomp_ to 1.5 × 10^–3^ but the resulting complex is not emissive in RT solution.^52^ In 2023, we reported that replacing Cp with Cp* resulted in a complex (^**Ph**^ [Cp*Ti]­CuBr in Figure 1), that is both emissive in RT THF solution (λ_max_ = 693 nm, Φ_PL_ = 1.3 × 10^–3^, τ = 0.18 μs) and relatively photostable (Φ_decomp_ = 1.5 × 10^–2^).^53^ One hypothesis for the enhanced emission is that steric constraint, namely that caused by Me-Me contacts between the two Cp* ligands and contacts between the Cp* ligand and the phenylethynyl ligands, restricts excited-state geometric distortion. To test this hypothesis, here we report the synthesis and photophysical properties of a titanocene derivative with *o*-xylylethynyl ligands (^**xyl**^ [Cp*Ti]­CuBr in Figure 1), to increase the steric congestion between the arylethynyl ligands and the Cp* ligands. X-ray diffraction data demonstrates that ^**xyl**^ [Cp*Ti]­CuBr is sterically congested, and we report that the complex is emissive in RT fluid solution (λ_max_ = 734 nm) with a photoluminescent quantum yield and lifetime approximately 10-fold greater (Φ_PL_ = 1.2 × 10^–2^, τ = 1.6 μs) than ^**Ph**^ [Cp*Ti]­CuBr. We also demonstrate that this complex sensitizes ^1^O_2_ formation and photon upconversion.

**Figure 1:**
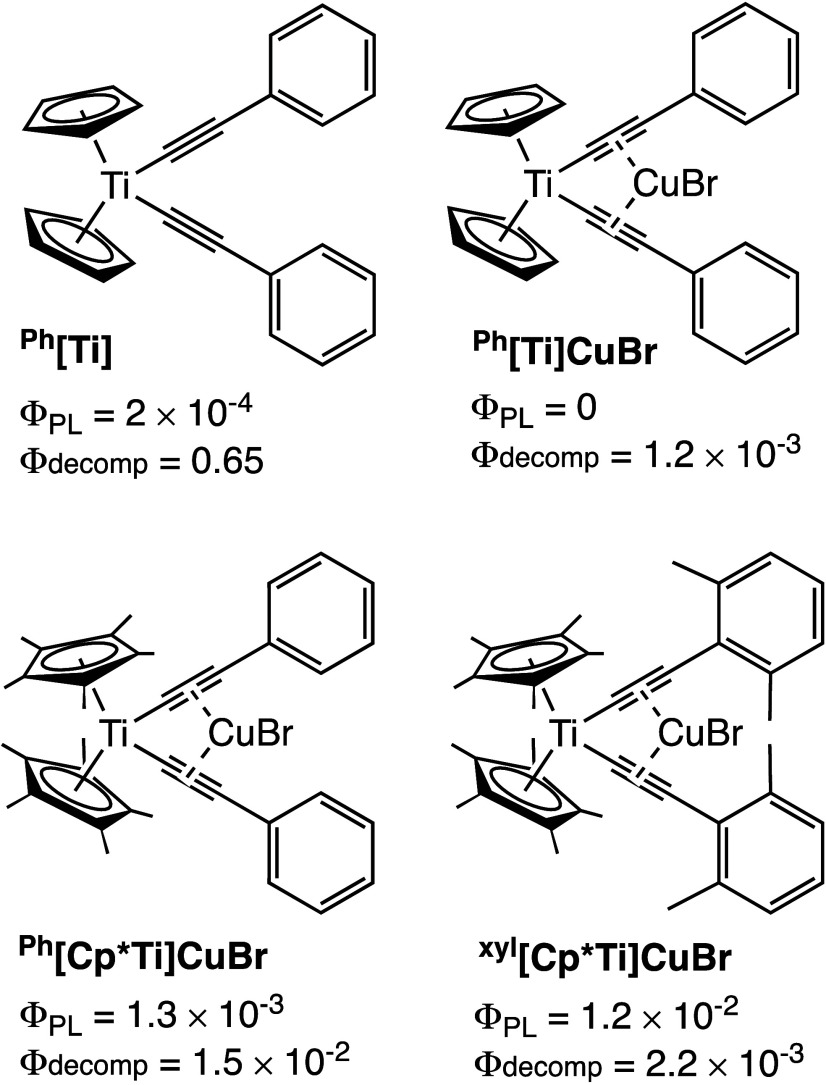
Arylalkynyl titanocenes investigated for emission in RT fluid solution. The values for Φ_PL_ and Φ_decomp_ for ^**Ph**^[Ti],^52^ ^**Ph**^ **[Ti]­CuBr**,^52^ and ^**Ph**^ [Cp*Ti]­CuBr^53^ were reported in deaerated THF. The data for ^**xyl**^ [Cp*Ti]­CuBr (in dearated THF) are reported herein.

## Experimental Section

### Materials and Methods

The complexes ^**Ph**^ **[Ti]­CuBr** ^52^ and ^**Ph**^ [Cp*Ti]­CuBr^53^ were prepared according to literature procedures. Bis­(pentamethylcyclopentadienyl)­titanium dichloride was obtained from Acros Organics; 2-ethynyl-1,3-dimethylbenzene was obtained from AA Blocks. ^1^H and ^13^C NMR spectra were recorded using a JEOL-500 spectrometer. Infrared spectra were recorded using a PerkinElmer Spectrum Two FT-IR spectrometer with a UATR attachment. Poly­(methyl methacrylate) (PMMA) films were prepared by dissolving 100 mg of PMMA (*M*_W_ ∼ 120,000) into 1 mL of CH_2_Cl_2_ by stirring for 1 h. After addition of 5 mg of the appropriate analyte, the solutions were stirred an additional 10 min. Films were prepared by drop-casting this solution onto quartz slides followed by curing for 24 h under ambient conditions. Photodecomposition quantum yields (Φ_decomp_) were performed using the method previously published for the ^**Ph**^ [Cp*Ti]­CuBr using a 428 nm diode laser (RMPC Laser) as the excitation source,^53^ and are detailed in the Supporting Information. Elemental analyses were performed by Midwest Microlabs.

### Steady-State Absorption and Emission

UV–vis absorption spectra were recorded using a Cary-50 spectrophotometer. Emission spectra were recorded using a Horiba Fluorolog-3 Spectrofluorometer equipped with either a FL-1013 liquid nitrogen dewar assembly or J-1933 solid-sample holder. All emission spectra were corrected for the response factor of the R928 photomultiplier tube. Relative solution-state photoluminescence quantum yields for ^**xyl**^ [Cp*Ti]­CuBr in tetrahydrofuran (THF) were determined using a [Ru­(bpy)_3_]^2+^ standard in air-saturated CH_3_CN (Φ_PL_ = 0.018).^54^ These solutions were absorbance matched at the excitation wavelength (450 nm), and the reported quantum yields are averages of at least three replicates. Emission spectra in RT solution were additionally corrected with blank subtraction. Singlet-oxygen emission was detected using a liquid-nitrogen cooled InGaAs detector (Horiba DSS-IGA020L).

### Time-Resolved Emission

Emission lifetimes were measured using a Photon Technology International (PTI) GL-3300 pulsed nitrogen laser operating at 3 Hz fed into a PTI GL-302 dye laser as the excitation source (Exciton PBD dye, 367 nm). At this wavelength and repetition rate, the dye laser output is rated at 200 μJ/pulse with an approximate spot size of 2 mm. The resulting data set was collected on an OLIS SM-45 EM fluorescence lifetime measurement system using a Hamamatsu R928 photomultiplier tube fed into a LeCroy Wavejet 352A oscilloscope through a Thorlabs VT2 variable resistance feed-through terminator. The monochromator was set to the emission wavelength and a 450 nm long-pass filter was also inserted before the PMT. All RT decay traces were fit to a single-exponential with background function (*I­(t) = I0 + Ae* ^*–k(t‑t0)*^) using OLIS Spectral Works. For 77 K measurements on ^**xyl**^ [Cp*Ti]­CuBr, the decay trace was fit to a double-exponential with x-offset function using IGOR Pro 9. No constraints or initial guesses were supplied. The fits and range over which the fits were performed are shown in the Supporting Information.

### Intensity-Dependent Emission Measurements

Intensity-dependent emission measurements on solutions containing 9,10-diphenylanthracene (**DPA**) and ^**xyl**^ [Cp*Ti]­CuBr or platinum­(II) octaethylporphyrin (**PtOEP**) were recorded on an Edinburgh FLS980 fluorescence spectrometer. Solutions containing 0.1 mM TTA-UC sensitizer (^**xyl**^ [Cp*Ti]­CuBr or **PtOEP**) and 10 mM **DPA** in THF were deaerated with nitrogen for 20 min prior to the measurement. The solution was excited by a 532 nm laser (Aixiz, AD-532–400T) passed through a 2 mm diameter iris (Newport ID-1.0) with a laser intensity ranging from 4.0 mW cm^–2^ to 47.0 W cm^–2^ and the emission was recorded by the fluorometer noted above. The intensity of the excitation source was controlled using the internal, variable neutral density filter of the Edinburgh FLS980 TCSPC accessory and measured using a power meter (Ophir Vega, 7Z01560) with a high-sensitivity power sensor (Ophir, 3A-FS 7Z02628).

### Ultrafast Transient Absorption

Ultrafast transient-absorption measurements were acquired on a previously described spectrometer.^55^ Briefly, a commercially available spectrometer (Helios FIRE, Ultrafast Systems) coupled to a 1 kHz Ti:sapphire amplifier (Astrella Coherent Systems) was used to acquire all data. The 800 nm fundamental was used to generate the 570 nm pump (100 μm beam size, 5 μJ/pulse, 150 fs fwhm, as determined using a Thorlabs BC106N CCD camera beam profiler) with an optical parametric amplifier and the visible probe through white light generation via a CaF_2_ crystal. Five mm quartz cuvettes containing ∼10 μM (A_570_ = 0.025) solutions of the complex in THF were bubble deaerated with N_2_ for 20 min prior to the measurements. Spectra and kinetics were averaged for two seconds at each delay time and two total scans were averaged together to generate the entire data set. Data were processed using the SurfaceXplorer software suite by first performing a background subtraction to remove any artifacts or laser scatter at any times, *t* < 0. This was followed by cropping the data set to remove areas outside of the temporal and spectral response of the probe and applying polynomial chirp correction Fits to the rise at 650 nm (Figure S10) were performed using an x-offset single-exponential function (*ΔOD­(t) = ΔOD(0) + Ae* ^*–(t‑t0)/τ)*^ *)* within the Igor Pro 9 software. Fits were performed on all data between the first and peak data points with no constraints or initial guesses supplied.

### Nanosecond Transient Absorption

Nanosecond transient absorption (ns-TA) data were acquired at room temperature using a commercially available spectrometer (LP980-KS, Edinburgh Instruments Ltd., Livingston, U.K.). Laser excitation of 570 nm (30 mJ/pulse, 3–6 ns fwhm, 5 mm beam diameter, determined using a Thorlabs BC106N CCD camera beam profiler) was generated by a Nd:YAG laser (Continuum Surelite I, Amplitude, San Francisco, California) equipped with a second-harmonic generation crystal pumping a Horizons optical parametric oscillator (Horizons OPO, Amplitude, San Francisco, California). The probe light was generated by a 150 W pulsed xenon arc lamp perpendicular to the pump source. Single wavelength transient absorption kinetics were collected by a photomultiplier tube (PMT) detector (Hamamatsu R928) connected to a Tektronix model MDO3052 (200 MHz) digital oscilloscope with a detection monochromator bandwidth of 1 nm. Transient spectra were acquired by a gated intensified iCCD. The probe background was collected between two laser shots and subtracted from the signal. A total of 40 laser shots were used to acquire both transient kinetics and spectra. Quartz cuvettes (1 cm, FireflySci, Inc.) containing ∼10 μM (A_570_ = 0.05) solutions of the complexes in THF were bubble deaerated with N_2_ for 20 min prior to the measurements. All single-wavelength kinetics were fit as described above for the ultrafast transient absorption data.

### Temperature-Dependent Emission

Temperature-dependent emission measurements were performed using the Edinburgh Instruments LP980-KS spectrometer described above in Nanosecond Transient Absorption, but instead set to emission mode. Samples were excited with a 570 nm pump (A_570_ = 0.05) that was incident perpendicular to the detection path. A total of 40 laser shots were used to acquire both emission decay kinetics and spectra. Changes in temperature were achieved through a Polyscience 9101 Refrigeration Circulation Chiller (Polyscience) connected to the sample holder. Kinetics were acquired at a temperature range of 10–50 °C. Following equilibration of the Chiller bath to the designated temperature, a cuvette containing only solvent was placed in the sample holder. The equilibration of solvent only cuvette was monitored with a thermocouple and required ∼ 20 min to equilibrate. Subsequently, the sample was placed into the sample holder and allowed to thermally equilibrate for 30 min. Quartz cuvettes (1 cm, FireflySci, Inc.) containing ∼ 10 μM solutions of the complexes in THF were bubble deaerated with N_2_ for 20 min prior to the measurements. All single-wavelength kinetics were fit with a single-exponential decay function modified with an x-offset (*I­(t) = I0 + Ae* ^*–(t‑t0)/τ)*^ *)* within the Igor Pro 9 software. Fits were performed on all data between the first and last data points with no constraints or initial guesses supplied.

### Syntheses

Cp*_2_ *Ti­(C*_*2*_ *C*_*6*_ *H*_*4*_ *Me*_*2*_ *)*_*2*_, ^**xyl**^ [Cp*Ti] To an oven-dried, 50 mL, two-neck, round-bottom flask under a positive pressure of argon was added THF (20 mL) and 2-ethynyl-1,3-dimethylbenzene (0.32 mL, 2.3 mmol, 3.0 equiv). After the pale-brown solution was cooled in a dry ice/acetone bath for 10 min, *n*-butyllithium (1.0 mL, 2.5 M, 2.5 mmol, 3.3 equiv) was added. After stirring for another 10 min, the flask was removed from the cooling bath and stirred for an additional 10 min. Cp*_2_TiCl_2_ (300 mg, 0.771 mmol, 1.0 equiv) was added and the mixture was stirred at room temperature in the absence of light for 4 h. The reaction mixture was concentrated to a red oil under reduced pressure and loaded onto a silica gel column (2.5 × 15 cm) and eluted using a 5% solution (v/v) of triethylamine in CH_2_Cl_2_. The solvent was removed under reduced pressure. *n*-pentane was added, and the flask was briefly sonicated in a cleaning bath. The solid was collected by filtration. The residue in the original flask was similarly treated with a second aliquot (1.5 mL) of *n*-pentane to achieve quantitative transfer. The solid was dried under vacuum yielding a red solid (329 mg, 74%). UV–Vis (THF) λ_max_ (ε); 535 sh (1130), 462 (2100), 319 sh (19600). ^1^H NMR (500 MHz, CDCl_3_) δ 6.98 (m, 6H), 2.34 (s, 12H), 2.03 (s, 30H); ^13^C {^1^H} NMR (125 MHz, CDCl_3_) δ 171.1, 137.1, 126.8, 126.4, 124.9, 123.3, 120.4, 22.1, 13.4. Anal. Calcd (found) for C_40_H_48_Ti• 1/2 H_2_O C, 82.03 (81.62); H, 8.43 (8.50). IR (neat, ATR) ν_C≡C_ not observed.

Cp*_2_ *Ti­(C*_*2*_ *C*_*6*_ *H*_*4*_ *Me*_*2*_ *)*_*2*_ *CuBr*, ^**xyl**^ [Cp*Ti]­CuBr To an oven-dried, 25 mL, two-neck round-bottom flask under a positive pressure of argon was added Cp*_2_Ti­(C_2_PhMe_2_)_2_ (50.0 mg, 0.0867 mmol, 1.0 equiv) and CuBr (24.9 mg, 0.173 mmol, 2.0 equiv). To the round-bottom flask was added CH_2_Cl_2_ (8 mL) and the mixture was stirred at room temperature in the absence of light for 4 h. To remove excess CuBr, the reaction mixture was filtered through Celite using CH_2_Cl_2_. The solvent was removed from the filtrate under reduced pressure. Minimal CH_2_Cl_2_ (0.25–0.50 mL) was added to wet the sample. *n*-pentane (10 mL) was added and the sample was sonicated in a cleaning bath to bring the sample off of the flask walls. The purple solid was collected using vacuum filtration and washed with *n*-pentane (10 mL). The solid was dried under vacuum (49.3 mg, 79%). UV–Vis (THF) λ_max_ (ε); 542 (4240), 488 (4640), 400 (5620), 334 sh (11500). ^1^H NMR (500 MHz, CDCl_3_) δ 7.06 (m, 6H), 2.62 (s, 12H), 2.06 (s, 30H); ^13^C {^1^H} NMR (125 MHz, CDCl3) δ 150.3, 139.1, 135.7, 128.1, 127.5, 123.7, 123.2, 23.9, 13.5. Anal. Calcd (found) for C_40_H_48_TiCuBr: C, 66.71 (67.14); H, 6.72 (6.73). IR (neat, ATR) ν_C≡C_ 1978 cm^–1^.

### Computational Methods

Gaussian 16 ^56^ was used for all DFT and TDDFT calculations. For each computational model, the geometry was optimized and the structure checked to be a minimum based on the frequency calculation. GaussView, version 6.32 ^57^ was used for all orbital imaging. Mulliken population analysis was performed using GaussSum3.^58^ Geometry optimization and TDDFT were performed using the functional B3LYP^59^ and the basis set 6–311+G­(d).^60^ Because all spectroscopic data reported herein are recorded in THF or 2-methyltetrahydrofuran, all calculations employed a Tomasi polarizable continuum model assigned the dielectric constant for THF.^61^

### X-ray Crystallography

Crystals of ^**xyl**^ [Cp*Ti]­CuBr were grown by vapor diffusion of Et_2_O into a solution of the complex in THF with 5% triethylamine (v/v). Single-crystal X-ray diffraction data were collected at 130 K using a Bruker D8 Quest diffractometer with Mo Kα radiation (λ = 0.71073 Å) and a Photon 3 detector. Data collection, data processing (SAINT), scaling, and absorption correction (SADABS, multiscan) were performed using the Apex 3 software suite.^62^ Space group determination (XPREP), structure solution by intrinsic phasing (SHELXT), and structure refinement by full-matrix least-squares techniques on *F* ^2^ (SHELXL) were performed using the SHELXTL software package.^63^ All non-hydrogen atoms were refined anisotropically. Hydrogen atoms attached to carbon atoms were placed in calculated positions using appropriate riding models. Crystallographic data are provided in the Supporting Information, Table S1. Crystallographic data are available in CIF form through the Cambridge Crystallographic Data Centre, CCDC deposition number 2440925.

## Results and Discussion

### Synthesis and Characterization

All syntheses were performed under an inert atmosphere, but the titanocene products are air-stable and can be handled under low-light, ambient conditions. The procedure for the synthesis of ^**xyl**^ [Cp*Ti] follows that of the corresponding synthesis of ^**Ph**^ [Cp*Ti], where the alkyne is deprotonated with BuLi and then treated with Cp*_2_TiCl_2_ (Figure 2).^53^ Purification by silica-gel column chromatography (5% Et_3_N in CH_2_Cl_2_, v/v) followed by precipitation from *n*-pentane yielded analytically pure samples with an NMR spectrum consistent with the structure (Figure S1). Likewise, the synthesis of ^**xyl**^ [Cp*Ti]­CuBr followed that for ^**Ph**^ [Cp*Ti]­CuBr, where the parent arylalkynyltitanocene is treated with CuBr (Figure 2).^53^ However, for the synthesis of ^**xyl**^ [Cp*Ti]­CuBr, this reaction was performed in CH_2_Cl_2_. Using THF (the solvent used successfully for ^**Ph**^ [Cp*Ti]­CuBr), resulted in mixtures of the ^**xyl**^ [Cp*Ti] starting material and the CuBr coordinated product that we were unable to separate, whereas using CH_2_Cl_2_ resulted in pure ^**xyl**^ [Cp*Ti]­CuBr. Removal of excess CuBr by filtration through Celite followed by precipitation from CH_2_Cl_2_ using *n*-pentane resulted in analytically pure material with an NMR spectrum consistent with the structure (Figure S2).

**Figure 2:**
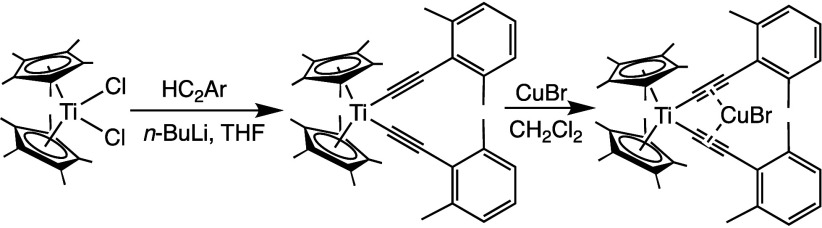
Synthesis of ^**xyl**^ [Cp*Ti] (HC_2_Ar = 2-ethynyl-1,3-dimethylbenzene) and ^**xyl**^ [Cp*Ti]­CuBr.

Crystals of ^**xyl**^ [Cp*Ti]­CuBr were grown by vapor diffusion of Et_2_O into a solution of the complex in THF with 5% triethylamine (v/v). The ^**xyl**^ [Cp*Ti]­CuBr complex crystallizes in the monoclinic space group *P*2_1_/*n*, with Z = 4 (Figure 3, Table S1, Figures S3–S5). The bromine atoms participate in C–H···Br interactions (H···Br = 2.965 Å; C–H···Br = 146.6°) that form dimers of complexes, where there is also some offset pi-stacking overlap (plane-to-plane distance of 3.096 Å) in the participating ligands (Figures S3, S4). The arylethynyl ligands create a binding pocket for CuBr through their coordination to Ti (Ti–C = 2.105(2) Å and 2.115(3) Å) having a C–Ti–C bond angle of 89.62(9)° (Figure 3). The C≡C bond lengths (1.223(3) Å and 1.228(3) Å), are similar to those of ^**Ph**^ [Cp*Ti]­CuBr. The angles about the C≡C bond, namely Ti–C≡C (174.8(2)° and 172.7(2)°) and C≡C–C (168.4(2)° and 168.9(3)°) deviate slightly from linearity. The additional methyl substituents in the xylylethynyl complex significantly impact the structure relative to the corresponding phenylethynyl derivative. Most notably, whereas the Ti–Cu–Br angle is 180° for ^**Ph**^ [Cp*Ti]­CuBr, that angle is bent (154.6(2)°) for the xylylethynyl complex, likely influenced by the more sterically encumbered space between the alkynyl arms created by the inner methyl groups of ^**xyl**^ [Cp*Ti]­CuBr. Furthermore, the Cp* ligands are inclined further from the Ti-alkyne-Cu plane for the xylylethynyl complex, resulting in a smaller centroid-Ti-centroid angle for ^**xyl**^ [Cp*Ti]­CuBr (139.2°) than for ^**Ph**^ [Cp*Ti]­CuBr (141.6°). Likewise, the average Ti-centroid distance for the xylylethynyl complex is elongated (2.10 Å) vs that of the phenylethynyl complex (2.04 Å). Such distortions are likely due to a balance of minimizing Me-Me contacts between the Cp* ligands^64^ and Me-Me contacts between the Cp* and xylylethynyl ligands and may serve to further restrict excited-state distortions in the xylylethynyl complex.

**Figure 3:**
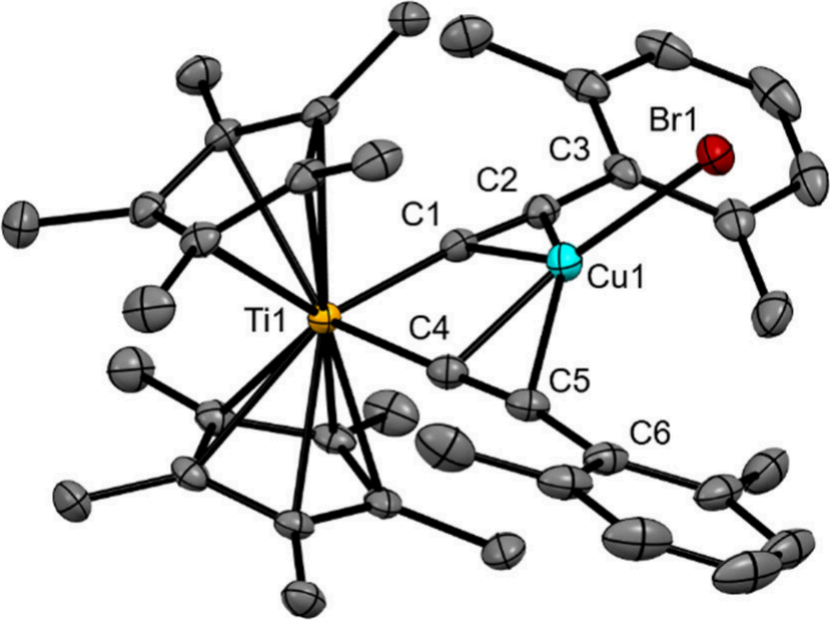
X-ray crystal structure of ^**xyl**^ [Cp*Ti]­CuBr shown as 50% probability ellipsoids. Hydrogen atoms have been omitted for clarity.

### Photophysics and Photochemistry

#### Emission and Absorption Spectra

The UV–vis absorbance spectrum of ^**xyl**^ [Cp*Ti]­CuBr reveals absorption with reasonably high molar absorptivity (≈ 5000 M^–1^ cm^–1^) throughout much of the visible region (Figure 4). This xylylethynyl complex is emissive in deaerated, room-temperature THF solution: λ_max_ = 734 nm, Φ_PL_ = 0.012, τ = 1.6 μs (Figure 4, Figure S6). The excitation spectrum closely matches the UV–vis absorption spectrum (Figure S7) demonstrating that emission is not due to an impurity. The quantum yield for photodecomposition (Φ_decomp_) for ^**xyl**^ [Cp*Ti]­CuBr in deaerated THF solution at RT is 2.2 × 10^–3^ and increases slightly (to 3.0 × 10^–3^) in air-saturated solution. Thus, this xylylethynyl complex is both more photostable than the corresponding phenylethynyl complex, ^**Ph**^ [Cp*Ti]­CuBr (Φ_decomp_ = 1.5 × 10^–2^),^53^ and has an emission lifetime and quantum yield an order of magnitude greater than those of the analogous phenylethynyl complex.

**Figure 4:**
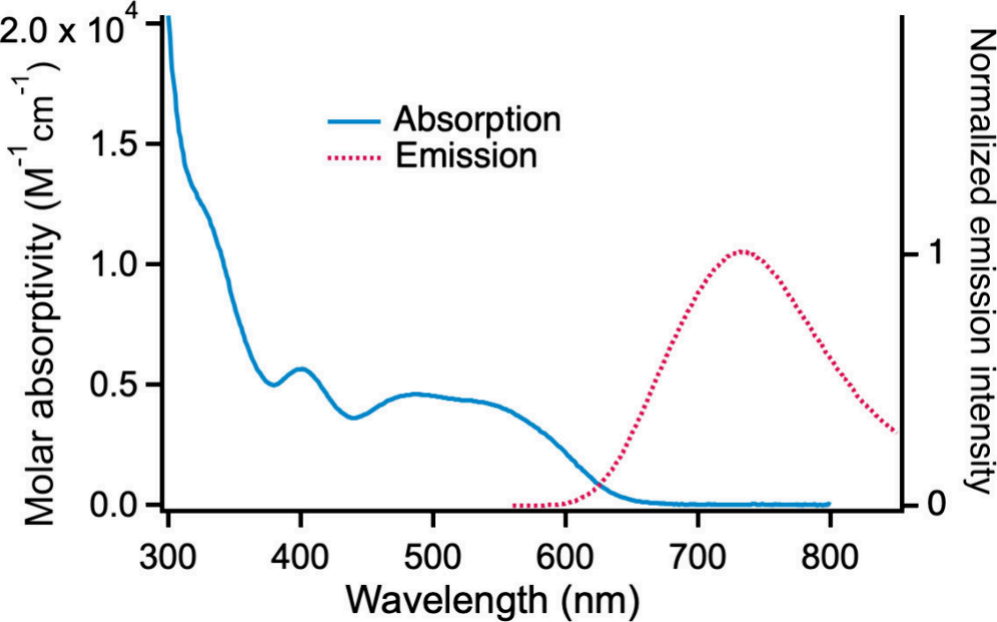
UV–vis absorption and emission spectra for ^**xyl**^ [Cp*Ti]­CuBr in THF solution at RT (λ_ex_ = 400 nm).

When previously rationalizing the photophysical impact of the replacement of Cp with Cp* (i.e., considering why ^**Ph**^ [Cp*Ti]­CuBr is emissive in RT fluid solution whereas ^**Ph**^ **[Ti]­CuBr** was not) several possible hypotheses were provided,^53^ including: (1) The additional steric bulk of the Cp* ligand results in an excited state that is less distorted than for the Cp complex; (2) Whereas for ^**Ph**^ **[Ti]­CuBr** the lowest-energy CT transition was dominated by MXMCT and phenylethynyl-to-Ti LMCT, the dominant orbitals involved for ^**Ph**^ [Cp*Ti]­CuBr changed to Cp*-to-Ti LMCT with no phenylethynyl-to-Ti LMCT; (3) The lowest-energy triplet (as measured by 77 K emission) is higher in energy for ^**Ph**^ [Cp*Ti]­CuBr than ^**Ph**^ **[Ti]­CuBr** and this may lead to a higher barrier for surface crossing for the former; and (4) Replacement of Cp with Cp* may lower the solvent reorganization by shielding dipole changes within the molecule. Many of the above hypotheses remain worthy of consideration in the context of ^**xyl**^ [Cp*Ti]­CuBr. Thus, to further elucidate the mechanism of this enhanced photophysics, additional spectroscopic and computational investigations were performed.

#### Temperature Dependence of Emission

The emission spectra for ^**xyl**^ [Cp*Ti]­CuBr were recorded at temperatures between 0 and 50 °C (Figure S8). Changing the temperature has no impact on the spectral profile or energy of the emission features but the emission intensity decreases at elevated temperatures. Likewise, emission decay at 700 nm exhibits single exponential kinetics (Figure S8) that decrease in lifetime (τ) with increasing temperature (Table S2). These decay rate constants (*k* = 1/τ) exhibit a linear Arrhenius relationship (Figure S9), yielding an activation barrier of 23 kJ mol^–1^. The temperature independence of the emission profile, the linear Arrhenius relationship, the oxygen sensitivity, and relatively long lifetime are all consistent with a phosphorescent emission mechanism.

#### Transient Absorption

Furthering the investigation into the excited-state dynamics, we performed transient-absorption measurements (Figures 5 and S10). On the nanosecond time scale (Figure 5), excitation into the LMCT absorption band at 570 nm yields a spectrum that is dominated by excited-state absorption features at 370, 450, and 650 nm. Single-wavelength kinetics at the maximum of each excited-state absorption peak exhibit single-exponential behavior with lifetimes of ∼ 1 μs (Table S3). All features are generated within the instrument response function (∼10 ns) and decay to baseline in <10 μs at similar rates to that observed in time-resolved emission, indicating that 1) little to no photodecomposition occurs during these measurements and 2) excitation into the LMCT band yields formation of the T_1_ state within the 10 ns instrument response. On subnanosecond time scales (Figure S10), 570 nm excitation yields similar features to the longer time-scale data but with some subtle differences. While the ground-state bleach at 540 nm exponentially decays over the course of the measurement (250 fs – 8 ns), the excited-state absorption at 660 nm initially increases for the first 4 ps with rise time of approximately 600 fs and then decreases concurrent with the ground-state bleach. This spectral evolution could be attributed to solvent reorganization, or more likely, due to the lack of energy change, intersystem crossing (ISC) from the singlet to triplet excited state. If the latter, then the rate constant for ISC for this complex is on the order of 2 × 10^12^ s^–1^.

**Figure 5:**
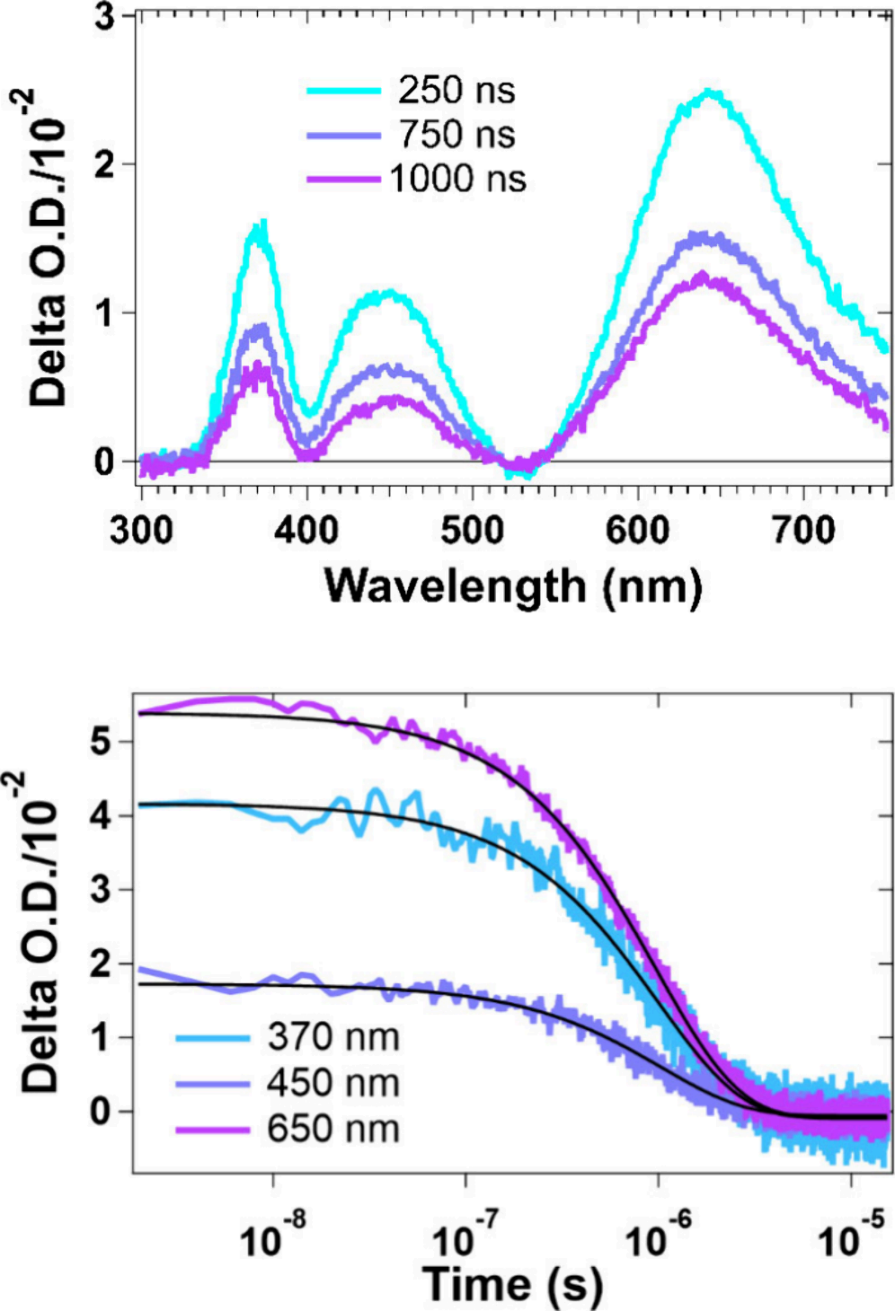
Transient absorption spectra (top) and single-wavelength kinetics (bottom) of ^**xyl**^ [Cp*Ti]­CuBr in THF solution at RT (λ_ex_ = 570 nm, A_570_ = 0.05).

#### Computational Analysis

Previous computational modeling of ^**Ph**^ [Cp*Ti]­CuBr was performed using the MN15/LANL2DZ//B3LYP/6–311+G­(d) (TDDFT//DFT) model^53^ that had been shown to accurately model the spectral properties of the Cp complexes, ^**R**^ **[Ti]­MX**.^52^ Therein, the charge-transfer component of the lowest-energy singlet and triplet for ^**Ph**^ [Cp*Ti]­CuBr was reported to be dominated by Cp*-to-Ti LMCT with some CuBr-to-Ti MXMCT character. No significant arylalkyne-to-Ti LMCT was evident.^53^ It was noted that the computationally modeled UV–vis spectrum was blue-shifted by approximately 2000 cm^–1^ relative to the experimental spectrum. It has now been determined that using B3LYP/6–311+G­(d) for both DFT and TDDFT provides a better model of the UV–vis spectra of these Cp* complexes. Thus, we report the TDDFT results for both ^**Ph**^ [Cp*Ti]­CuBr, and ^**xyl**^ [Cp*Ti]­CuBr using this model (Figure 6, Table 1, Charts S1 and S2). It is worth noting that when using a starting structure for ^**xyl**^ [Cp*Ti]­CuBr with a linear Ti–Cu–Br arrangement that was based on the structure of ^**Ph**^ [Cp*Ti]­CuBr, the linear Ti–Cu–Br arrangement is maintained upon minimization. However, when the X-ray crystal structure for ^**xyl**^ [Cp*Ti]­CuBr was used as the starting structure for the DFT calculation, the bent Ti–Cu–Br arrangement is maintained in the minimized structure. That bent structure has an energy that is 0.0033 hartree lower than the minimized linear structure and the DFT structure is in reasonable agreement with the crystal structure (Table S4). The TDDFT for ^**xyl**^ [Cp*Ti]­CuBr was based on the lower-energy “bent” DFT minimized structure.

**Figure 6:**
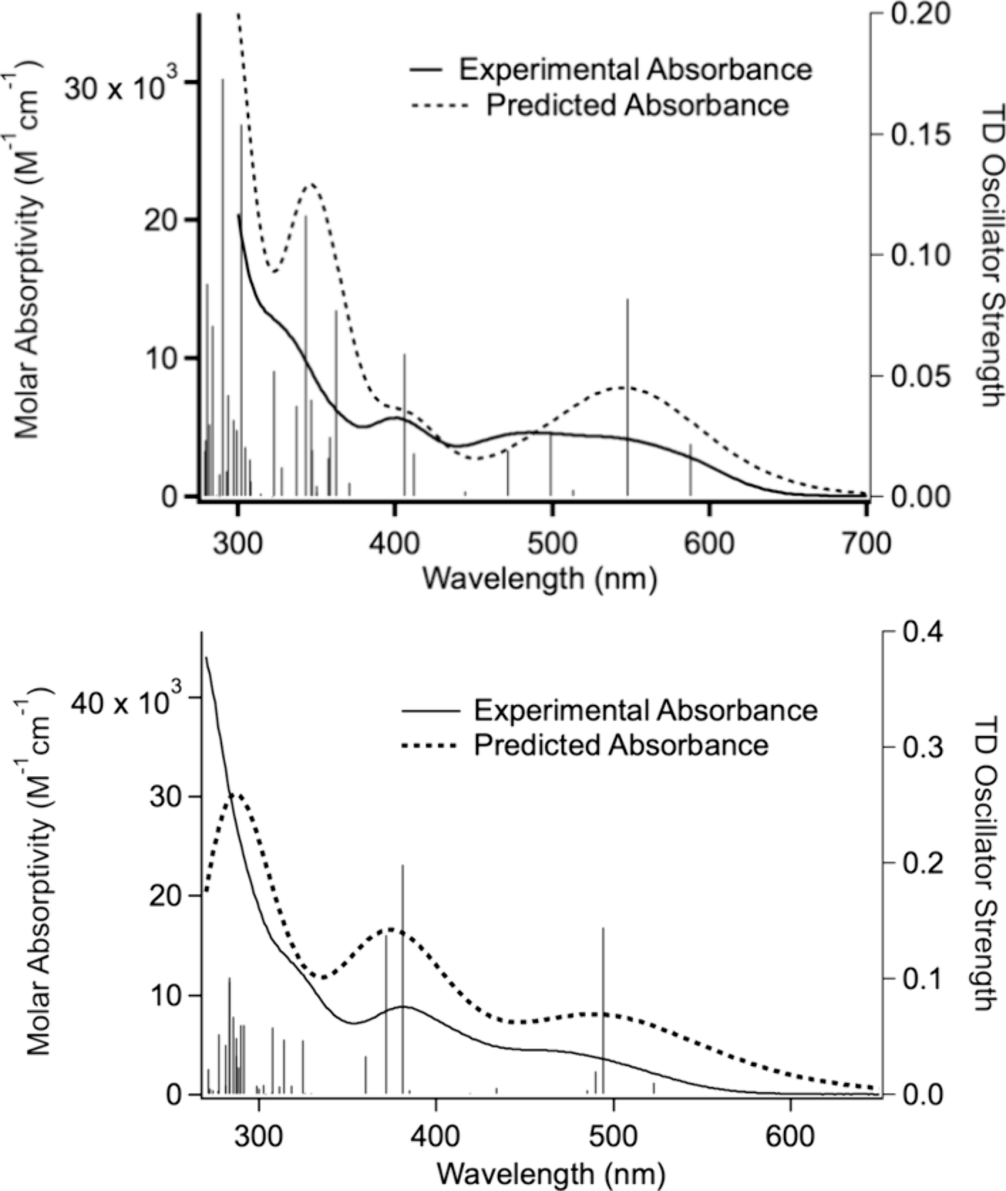
Overlay of experimental absorption spectra of ^**xyl**^ [Cp*Ti]­CuBr (top) and ^**Ph**^ [Cp*Ti]­CuBr (bottom) in THF solvent with the TDDFT predicted transitions and spectra. The HWHM for spectral prediction was chosen to best match the experimental spectrum: 1500 cm^–1^ for ^**xyl**^ [Cp*Ti]­CuBr, and 2500 cm^–1^ for ^**Ph**^ [Cp*Ti]­CuBr.

**Table 1:** Orbital Contribution and Population Analysis^a^ for Lowest-Energy Excited States

ES	dominant orbitals^b^	λ(nm), f^c^	Ti	Cp*	C_2_Aryl	CuBr
^xyl^[Cp*Ti]CuBr						
T_1_	HOMO → LUMO (96%)	660, 0	8 → 57 (49)	38 → 10 (−28)	41 → 27 (−14)	12 → 7 (−5)
S_1_	HOMO → LUMO (93%)	588, 0.0218	8 → 57 (49)	38 → 10 (−28)	40 → 27 (−13)	14 → 7 (−7)
S_2_	HOMO–1 → LUMO (89%)	548, 0.0819	1 → 57 (56)	11 → 10 (−1)	49 → 27 (−22)	38 → 7 (−31)
^**Ph**^ [Cp*Ti]CuBr						
T_1_	HOMO → LUMO (80%)	577, 0	3 → 50 (47)	27 → 8 (−19)	39 → 35 (−4)	31 → 7 (−24)
S_1_	HOMO–1 → LUMO (78%)	523, 0.0104	10 → 50 (40)	55 → 8 (−47)	21 → 35 (14)	14 → 7 (−7)
S_2_	HOMO → LUMO (69%)	494, 0.1444	4 → 50 (46)	32 → 8 (−24)	36 → 35 (−1)	27 → 7 (−20)

a Mulliken population analysis was performed using GaussSum3.^58^

b Dominant orbitals involved in each transition along with the percent contribution. A complete list of orbital pairs for these transitions is included in the Supporting Information (Charts S1 and S2) along with those for all transitions lower in energy than 300 nm (including oscillator strengths and coefficients for each orbital pair).

c Wavelength and oscillator strength for each transition.

For each complex, the orbital contribution for the lowest-energy triplet (T_1_), and the two lowest-energy singlets (S_1_ and S_2_) are presented along with the Mulliken population analysis (Table 1, Figure 7). In both complexes, S_2_ has the higher oscillator strength, making the largest contribution to the lowest-energy absorption band (Figure 6). For ^**Ph**^ [Cp*Ti]­CuBr, this model still predicts the charge-transfer component of T_1_ is dominated by a mixture of Cp*-to-Ti LMCT and CuBr-to-Ti MXMCT but the degree of Cp*-to-Ti LMCT is diminished relative to that predicted by TDDFT using MN15/LANL2DZ. Of the two lowest-energy singlets, S_2_ most closely mirrors these orbital contributions. In contrast, for ^**xyl**^ [Cp*Ti]­CuBr, T_1_ is comprised of significantly more C_2_Aryl-to-Ti and Cp*-to-Ti LMCT character and significantly less CuBr-to-Ti MXMCT character; and in this case, S_1_ most closely mirrors the orbital contributions of T_1_. The larger contribution of C_2_Aryl-to-Ti LMCT character for ^**xyl**^ [Cp*Ti]­CuBr may be a consequence of the contribution of the methyl substituents to the electron density of the aryl ring. However, it is also possible that the restricted orientation of the aryl rings in the xylylethynyl complex impacts the electronic structure. For example, for ^**Ph**^ **[Ti]­CuBr**, different rotational orientations of the phenyl ring were shown to impact the calculated UV–vis spectrum.^51^ It is also worth noting that for both complexes, charge transfer to Ti appears to account for only about half of the overall transition, suggesting significant π-π* character, which appears to be largely centered on the C_2_Aryl ligand.

**Figure 7:**
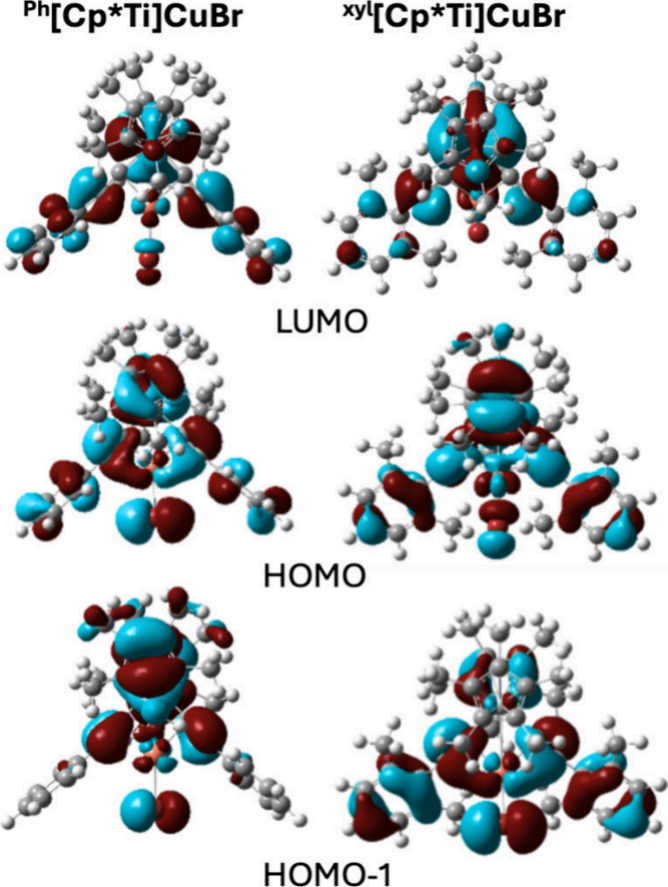
Frontier orbitals (isovalue = 0.02) for ^**Ph**^ [Cp*Ti]­CuBr (left), and ^**xyl**^ [Cp*Ti]­CuBr (right) at the B3LYP/6–311+G­(d) level of theory.

#### Emission in Rigid Matrices

Solvating molecules in a poly­(methyl methacrylate) (PMMA) film is a common strategy to investigate the impact of environmentally controlled rigidification on molecular photophysics.^55,65–69^ Whereas ^**Ph**^ **[Ti]­CuBr** is not emissive in solution at RT, emission is observed in PMMA films at RT.^53^ Emission in PMMA film at RT has also been investigated for ^**Ph**^ [Cp*Ti]­CuBr, and ^**xyl**^ [Cp*Ti]­CuBr (Figure 8, Table 2). Under these conditions, ^**Ph**^ **[Ti]­CuBr**, ^**Ph**^ [Cp*Ti]­CuBr, and ^**xyl**^ [Cp*Ti]­CuBr have similar phosphorescent lifetimes (between 3.5 and 5.8 μs, Table 2 and Figure S11). This suggests that the difference of the orbitals involved in the excited state for these complexes does not significantly impact the emission lifetime, rather the data is consistent with the rigid, PMMA environment suppressing nonradiative decay, which in solution is achieved through intramolecular steric hindrance resulting in increased excited-state lifetimes through this series. Notably, the phosphorescent lifetime of the xylylethynyl complex in solution is approximately 1/4 of that in PMMA film, suggesting the intrinsic molecular rigidification limit is being approached.

**Figure 8:**
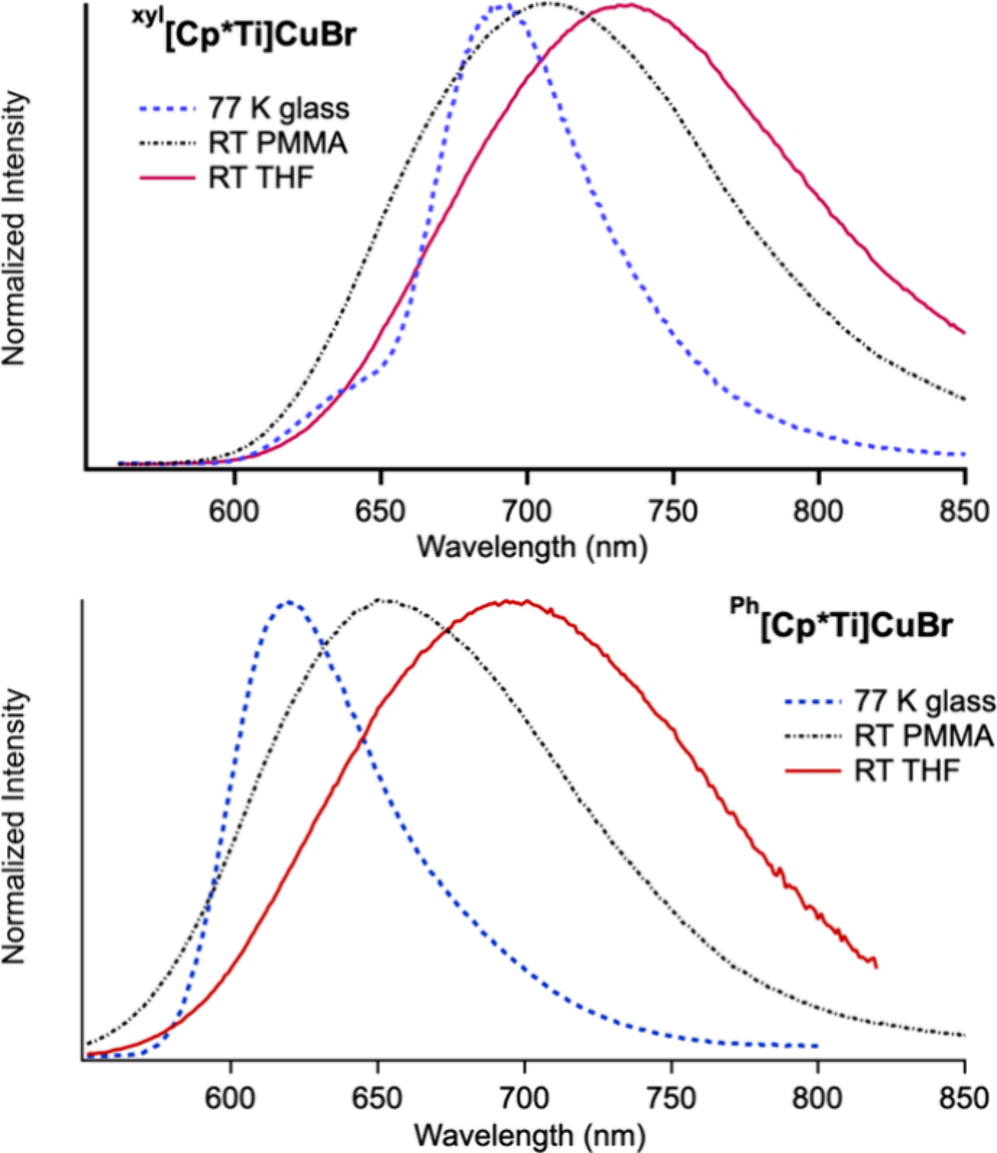
Comparison of emission spectra in THF solution and PMMA films at room temperature and 2-MeTHF glass at 77 K for ^**xyl**^ [Cp*Ti]­CuBr (top) and ^**Ph**^ [Cp*Ti]­CuBr (bottom).

**Table 2:** Emission Wavelengths and Lifetimes for the Complexes in Various Media

	RT THF soln	RT PMMA film^a^	77 K glass
^**Ph**^ **[Ti]CuBr**	Not emissive	723 nm (5.8 μs)^b^	715 nm (35.8 μs)^c^
^**Ph**^ [Cp*Ti]CuBr	693 nm (0.18 μs)^c^	650 nm (3.5 μs)	616 nm (655 μs)^d^
^**xyl**^ [Cp*Ti]CuBr	734 nm (1.6 μs)	707 nm (5.6 μs)	692 nm (180 μs)^d^

a All samples were prepared using 5 wt % titanocene in PMMA.

b The emission spectrum and lifetime for ^**Ph**^ **[Ti]­CuBr** were reported in ref 53 as 4.6 μs under much higher loading (15% by weight).

c From ref 53.

d Weighted average from biexponential fit. See ref 55 for weighting functions.

The emission spectra and lifetimes at 77 K in 2-methyltetrahydrofuran (2-MeTHF) glass were also recorded (Figure 8, Table 2, Figure S12). Under these conditions, in addition to the effect of environmental rigidification, thermally activated crossing to the GS surface will also presumably be inhibited. The excited-state lifetimes increase with increasing emission energy, consistent with energy-gap-law behavior.^70^

#### Solution Phase Lifetime and Spectral Analyses

The observation that the room-temperature emission lifetime in solution for ^**xyl**^ [Cp*Ti]­CuBr is greater than that of ^**Ph**^ [Cp*Ti]­CuBr, despite having a lower emission energy, is not consistent with energy-gap law behavior. Furthermore, if the excited-state energy were impacting the lifetime through its effect on the relative energy of the potential-well surface crossing, then ^**xyl**^ [Cp*Ti]­CuBr would be expected to have the lower emission lifetime in solution. Thus, the data are consistent with hindered excited-state distortion being responsible for the longer excited-state lifetimes in solution at room temperature.

If the excited-state distortion for ^**xyl**^ [Cp*Ti]­CuBr is lower compared to that of ^**Ph**^ [Cp*Ti]­CuBr, then the Stokes-Shift would be expected to be lower for the former.^4^ A true Stokes shift would require observation of both absorption into, and emission from the triplet state. However, the absorption spectrum represents spin-allowed absorptions into the singlet states. Regardless, the difference in energy between the lowest-energy absorption and the phosphorescence maximum may serve as a surrogate for the relative magnitudes of the Stokes shift (assuming similar 0–0 energy gaps between the lowest-energy singlet and triplet for each complex). The energy difference between the lowest-energy absorption shoulder and emission maximum for ^**xyl**^ [Cp*Ti]­CuBr is 4200 cm^–1^ (Figure 4) compared with 6600 cm^–1^ for ^**Ph**^ [Cp*Ti]­CuBr (Figure S13), consistent with a smaller Stokes shift (and thus smaller excited-state distortion) for the xylylethynyl analogue.

Likewise, steric restriction may also be manifested in the spectral width of the phosphorescence in fluid solution; namely, a larger excited-state distortion should result in a greater full-width at half-maximum (fwhm) for phosphorescence.^4^ For ^**xyl**^ [Cp*Ti]­CuBr, the fwhm of the phosphorescence is 2600 cm^–1^, compared with 2900 cm^–1^ for ^**Ph**^ [Cp*Ti]­CuBr (Figure S14), again consistent with greater steric restriction in the xylylethynyl complex.

### Photosensitization

#### Photon Upconversion via Triplet–Triplet Annihilation (TTA-UC)

TTA-UC is a nonlinear optical process where the energy from two low-energy photons are combined to generate a higher-energy excited state.^71^ This process is of interest for a range of applications including anticounterfeiting,^72^ oxygen sensing,^73^ bioimaging,^74^ solar energy conversion,^75^ and more.^76,77^ TTA-UC is typically facilitated by combining an organic emitter with a sensitizing coordination complex containing heavy metals like iridium, ruthenium, and platinum. Here we probed ^**xyl**^ [Cp*Ti]­CuBr as an alternative, more earth-abundant sensitizer for TTA-UC by combining it with the prototypical emitter molecule 9,10-diphenylanthracene (**DPA**).^71^ Under 532 nm excitation, a THF solution containing **DPA** and ^**xyl**^ [Cp*Ti]­CuBr (10 mM: 100 μM) exhibits emission from 400 to 500 nm that is consistent with that for direct excitation of **DPA** at 350 nm (Figure 9a). In contrast, for the ^**xyl**^ [Cp*Ti]­CuBr solution emission is only observed above 600 nm­(i.e., phosphorescence from ^**xyl**^ [Cp*Ti]­CuBr). Intensity-dependent emission measurements for the **DPA** and ^**xyl**^ [Cp*Ti]­CuBr solution exhibits quadratic-to-linear like behavior^78^ with a threshold intensity (i.e., *I*_th_ value)^79^ of ∼250 mW/cm^2^ (Figure 9b). This value is comparable to that observed using the more common Pt­(II) octaethylporphyrin sensitizer under the same conditions (∼200 mW/cm^2^, Figure S15). But it is important to note that the measurement and data quality with the ^**xyl**^ [Cp*Ti]­CuBr containing solution is complicated by the photoinstability of the sensitizer under these high excitation power conditions. Consequently, we were required to replace the sample every 3–5 intensity steps. Nonetheless, these observations are consistent with a TTA-UC mechanism where excitation into the LMCT band of ^**xyl**^ [Cp*Ti]­CuBr is followed by triplet energy transfer to **DPA**, triplet–triplet annihilation, and emission from a singlet excited state of **DPA**.

**Figure 9:**
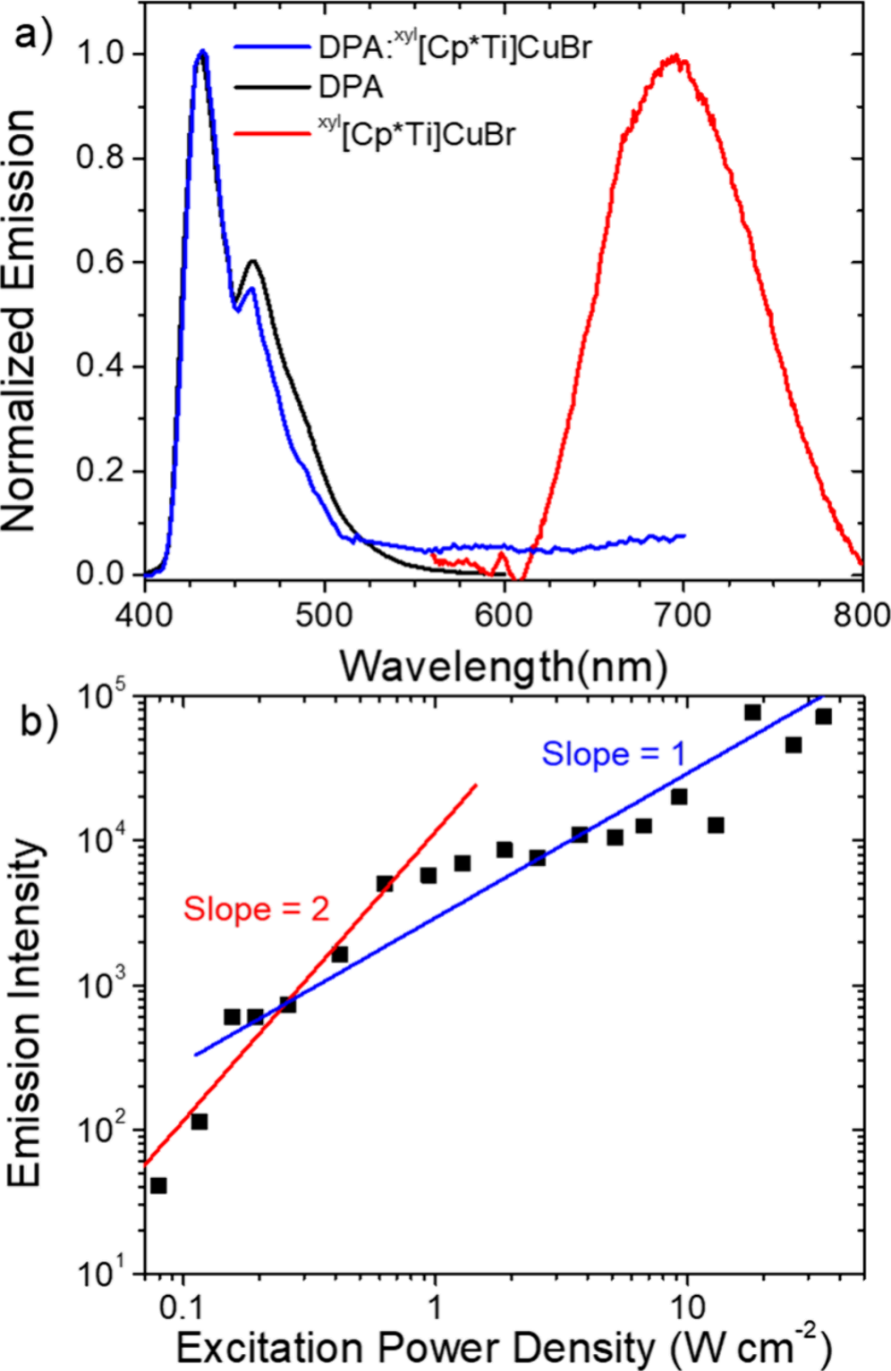
a) Normalized emission spectra for **DPA** (black, λ_ex_ = 350 nm), ^**xyl**^ [Cp*Ti]­CuBr (red, λ_ex_ = 532 nm, 47 W cm^–2^) and 10 mM:100 μM **DPA**:^**xyl**^ [Cp*Ti]­CuBr (blue, λ_ex_ = 532 nm) in nitrogen deaerated THF. b) Emission intensity for the same solution at 430 nm (black square) with respect to 532 nm excitation intensity. Lines with a slope of 1 (blue) and 2 (red) are overlaid on the data.

#### Singlet Oxygen Sensitization

Singlet oxygen photosensitizers are useful in a range of applications including photodynamic therapy, degradation of organic pollutants, and chemical synthesis.^80–84^ Transition-metal photosensitizers typically use second- and third-row transition metals because the higher degree of spin–orbit coupling in such sensitizers results in larger rate constants for intersystem crossing to the necessary triplet excited states.^85–87^ Herein we investigated ^**xyl**^ [Cp*Ti]­CuBr as an alternative, more earth-abundant sensitizer. The excited-state lifetime of ^**xyl**^ [Cp*Ti]­CuBr was measured in Ar-purged, air-saturated, and oxygen-saturated THF solution at RT. The lifetime decreases with increased oxygen concentration and a Stern–Volmer plot resulted in a value for the quenching rate constant, *k*_O2_, of 7 × 10^7^ M^–1^ s^–1^ (Table S5, Figure S16). This is within the range of rate constants for quenching by oxygen found for a series of zirconocenes ((5–18) × 10^7^ M^–1^ s^–1^).^38^ If such quenching results in ^1^O_2_, the characteristic NIR emission should be evident at 1270 nm. For these investigations, solutions of ^**xyl**^ [Cp*Ti]­CuBr in air-saturated CCl_4_ were analyzed because of the much longer lifetime of ^1^O_2_ in CCl_4_ (900 μs) than in THF (20 μs).^88^ An intense emission peak was detected at 1270 nm upon excitation at 550 nm (Figure 10), indicating the formation of ^1^O_2_. An excitation spectrum (λ_em_ = 1270 nm) closely matches the UV–vis spectrum of ^**xyl**^ [Cp*Ti]­CuBr in CCl_4_ (Figure 10) demonstrating that the xylylethynyl complex serves as the photosensitizer.

**Figure 10:**
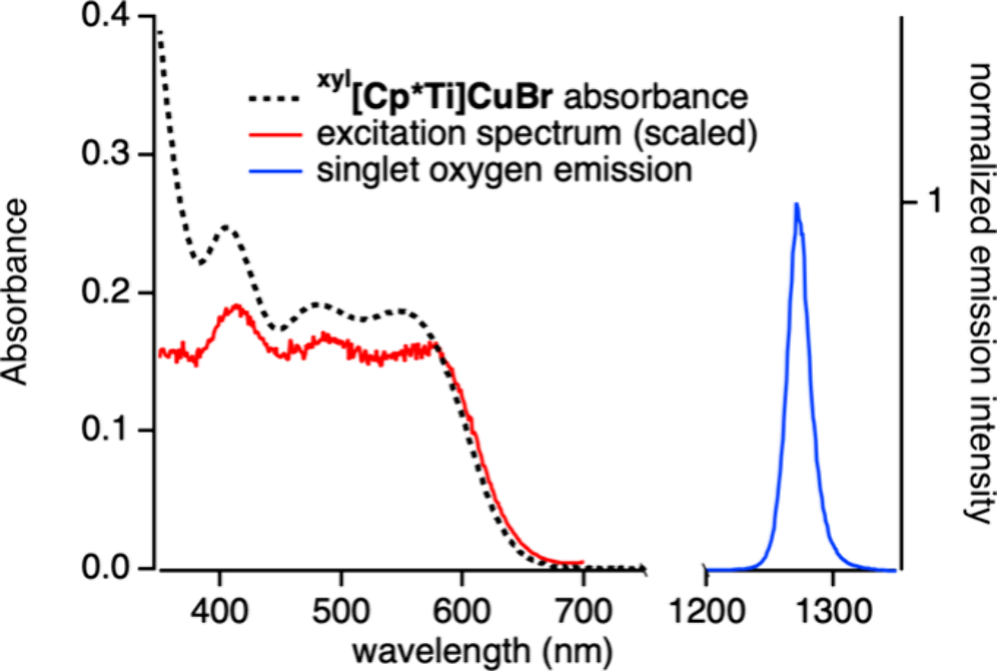
Emission spectrum of ^1^O_2_ (blue) in an air-saturated solution of ^**xyl**^ [Cp*Ti]­CuBr (5 × 10^–5^ M) in CCl_4_ (λ_ex_ = 550 nm). The corresponding scaled excitation spectrum (red, λ_em_ = 1270 nm) and absorption spectrum (black) were recorded on the same sample as the emission spectrum.

## Conclusions

The first Ti complex with a microsecond phosphorescence lifetime in room-temperature solution, ^**xyl**^ [Cp*Ti]­CuBr, is reported. Compared with related complexes, Φ_PL_ and τ increase in the order ^**Ph**^ **[Ti]­CuBr** ≪ ^**Ph**^ [Cp*Ti]­CuBr < ^**xyl**^ [Cp*Ti]­CuBr. Possible reasons for the enhanced photophysics have been discussed previously^53^ and include (1) restricted excited-state distortion due to added steric bulk, (2) changes in excited-state orbital character, (3) an increased excited-state energy lowers *k*_nr_ through energy gap law behavior and/or an increase in the activation barrier for excited-state to ground-state potential-well surface crossing, and (4) replacement of Cp with Cp* may diminish solvent reorganization by shielding the solvent from interactions with dipole changes in the molecule. The photoluminescence quantum yields and lifetimes increase with added steric bulk in support of hypothesis (1). Likewise, analyses of apparent Stokes-shifts and emission spectral widths of the two Cp* complexes in RT fluid solution are consistent with the xylylethynyl complex having a less-distorted excited state than the corresponding phenylethynyl complex, also in support of hypothesis (1). Second, despite significantly different excited-state orbital character, the three complexes have similar lifetimes in PMMA films. This is inconsistent with hypothesis (2). Third, the lifetime of ^**xyl**^ [Cp*Ti]­CuBr is approximately an order of magnitude higher than the corresponding phenylethynyl complex, despite having a lower emission energy. This is inconsistent with hypothesis (3). Lastly, the addition of the xylyl methyl groups may further shield the solvent from dipole changes in the molecule suggesting that hypothesis (4) may indeed contribute to the increased lifetime through decreased outer-sphere, excited-state reorganization. Thus, the data herein are consistent with differences in excited-state reorganization being responsible for increased lifetimes.

These investigations focused on the CuBr complexes for comparison to earlier work on ^**Ph**^ [Cp*Ti]­CuBr. Furthermore, earlier work on ^**R**^ **[Ti]­CuX** complexes demonstrated that the CuBr and CuCl complexes have nearly identical RT absorption spectra and nearly identical 77 K emission spectra. However, for ^**Ph**^ **[Ti]­CuX**, the complex with CuBr had significantly more CuX-to-Ti MXMCT character in the excited state than the CuCl complex. It would be worthwhile to investigate the CuCl and CuI complexes, not only due to the difference in electronegativity, but also due to the size differences of these copper halides.

It is noteworthy that the excited-state lifetime of ^**xyl**^ [Cp*Ti]­CuBr in THF solution exceeds that of [Ru­(bpy)_3_]^2+^ (650 ns) in deaerated aqueous solution,^89^ though its Φ_PL_ is less than that of [Ru­(bpy)_3_]^2+^ (0.063 in H_2_O).^54^ Furthermore, ^**xyl**^ [Cp*Ti]­CuBr sensitizes both ^1^O_2_ formation and photon upconversion. To our knowledge, these are the first demonstrations of a titanium complex sensitizing these processes. Such a development is significant due to the relative natural abundance of titanium versus ruthenium, with titanium being the second most abundant transition metal in the earth’s crust.^90^ One impediment to the use of ^**xyl**^ [Cp*Ti]­CuBr as a photosensitizer is its sensitivity to photodegradation. However, the above results indicate that titanium­(IV) complexes are a promising class of photosensitizers and are worthy of further investigation.

## Supplementary Material





## Notes

Details of photodecomposition quantum yield measurement; NMR spectra for complexes prepared herein; crystallographic data and supplemental figures for the solid-state structure of ^**xyl**^ **[Cp*Ti]­CuBr**; excitation spectrum and luminescence decay trace for ^**xyl**^ **[Cp*Ti]­CuBr** in THF; temperature dependent emission spectra and Arrhenius plot for ^**xyl**^ **[Cp*Ti]­CuBr**; transient absorption spectra and exponential decay lifetimes for the transient absorptions for ^**xyl**^ **[Cp*Ti]­CuBr**; Comparison of interatomic distances and angles for ^**xyl**^ **[Cp*Ti]­CuBr** obtained by DFT and X-ray diffraction; orbital contributions for lowest energy triplet and singlets for ^**xyl**^ **[Cp*Ti]­CuBr** and ^**Ph**^ **[Cp*Ti]­CuBr**; Luminescence decay traces and fits for ^**xyl**^ **[Cp*Ti]­CuBr**, ^**Ph**^ **[Cp*Ti]­CuBr**, and ^**xyl**^ **[Cp*Ti]­CuBr** and ^**Ph**^ **[Ti]­CuBr** in PMMA film. Luminescence decay traces and fits for ^**xyl**^ **[Cp*Ti]­CuBr**, and ^**Ph**^ **[Cp*Ti]­CuBr** in 77 K glass; Absorption and emission spectra for ^**Ph**^ **[Cp*Ti]­CuBr** in THF; Emission intensity of ^**xyl**^ **[Cp*Ti]­CuBr** and ^**Ph**^ **[Cp*Ti]­CuBr** vs energy (cm^–1^); Emission spectra and power dependence for photon upconversion with Pt­(OEP); Lifetime of ^**xyl**^ **[Cp*Ti]­CuBr** in THF at RT as a function of [O_2_], and Stern–Volmer plot. (PDF)
Cartesian coordinates for optimized structures of ^**xyl**^ **[Cp*Ti]­CuBr** and ^**Ph**^ **[Cp*Ti]­CuBr** using B3LYP/6–311+G­(d) (XYZ)
