# Supplementary material for: Xylylethynyl Titanocene with a Microsecond Emission Lifetime Photosensitizes Singlet-Oxygen Formation and Photon Upconversion
Source: Inorg Chem. 2025 Jul 17;64(29):14977–88. doi: 10.1021/acs.inorgchem.5c01773 (PMC12308804; doi:10.1021/acs.inorgchem.5c01773)
Supplement: Supplementary file 1 [file ic5c01773_si_001.pdf]

Supporting Information for:

Xylylethynyl titanocene with a microsecond emission lifetime photosensitizes singlet-oxygen formation and photon upconversion

Jack M. Sledesky,[a] John H. Zimmerman,[a] Henry C. London,[b] Ethan C. Lambert,[b] Colin D. McMillen,[c] Matilda Barker,[a] Kenneth Hanson,*,[b] Paul S. Wagenknecht*,[a]

[a] Department of Chemistry, Furman University, Greenville, SC 29609. [b] Department of Chemistry and Biochemistry, Florida State University, Tallahassee, FL 32306. [c] Department of Chemistry, Clemson University, Clemson, SC 29634.

* Kenneth Hanson: hanson@chem.fsu.edu *Paul S. Wagenknecht: paul.wagenknecht@furman.edu

**Contents**	**Pages**
Experimental details of quantum yield of decomposition measurements:	S2
^{1}H and ^{13}C spectra for ^{xyl}**[Cp*Ti]** and ^{xyl}**[Cp*Ti]CuBr**	S3 – S4
Crystallographic data for ^{xyl}**[Cp*Ti]CuBr**	S5
Supplementary crystallographic figures for ^{xyl}**[Cp*Ti]CuBr**	S6 – S7
Luminescence decay trace for ^{xyl}**[Cp*Ti]CuBr** in argon purged THF	S7
Absorption and excitation spectra for ^{xyl}**[Cp*Ti]CuBr** in THF solution	S7
Temperature dependence of emission spectra and kinetic traces for ^{xyl}**[Cp*Ti]CuBr**	S8
Temperature-dependent emission fit parameters and Arrhenius plot for ^{xyl}**[Cp*Ti]CuBr**	S8
Transient-absorption spectra for ^{xyl}**[Cp*Ti]CuBr** in THF	S9
Table of exponential-decay lifetimes for the fits to the excited state absorptions	S9
Key Interatomic Distances and Angles for ^{xyl}**[Cp*Ti]CuBr**	S9
Charts of orbital contributions to the key transitions for ^{xyl}**[Cp*Ti]CuBr** and ^{Ph}**[Cp*Ti]CuBr**	S10 – S15
Luminescence decay traces and fits in PMMA film and 77 K 2-MeTHF	S15
Absorption and emission spectra for ^{Ph}**[Cp*Ti]CuBr** in THF solution at RT	S16
Emission intensity of ^{xyl}**[Cp*Ti]CuBr** and ^{Ph}**[Cp*Ti]CuBr** vs energy (cm^{-1})	S16
Emission spectra and power dependence for photon upconversion with **PtOEP**	S17
Lifetime of ^{xyl}**[Cp*Ti]CuBr** in THF at RT as a function of [O_2] and Stern-Volmer plot	S18

Experimental details of quantum yield of decomposition measurements: A cuvet containing a THF solution of a known volume of the complex was irradiated using a 428 nm diode laser (RMPC Laser) set to a power of approximately 12 mW and passed through a 2 mm diameter iris. The contents of the cuvet were stirred continuously during the period of the photolysis (typically 20 minutes). Photon flux was determined from optical power which was measured using a Thorlabs S120VC photodiode connected to a PM100USB optical power and energy meter (factory calibrated annually). Initial concentrations were chosen to have an absorbance near 1.5 at the irradiation wavelength, and photolysis time was typically chosen to result in a decrease in absorbance of approximately 0.2 absorbance units, ensuring that the fraction of incident radiation absorbed is nearly constant over the course of the photolysis. UV-Vis spectra were recorded at 20.0 ˚C before and after photolysis. Concentration changes were determined by measuring the absorbance change at the lowest-energy absorbance maximum and dividing by the molar absorptivity. Moles of analyte that decomposed were determined from the change in concentration and the sample volume. The number of moles of photons absorbed was determined from the photon flux, the time of photolysis, and the fraction of light absorbed by the sample. Exhaustive photolysis showed that the decomposition products do not absorb at the wavelength of the LMCT band and thus no correction for product absorbance was necessary. All reported Φ_{decomp} values are averages of at least three replicates. Estimated error for $\Phi_{decomp} = \pm 20\%$.

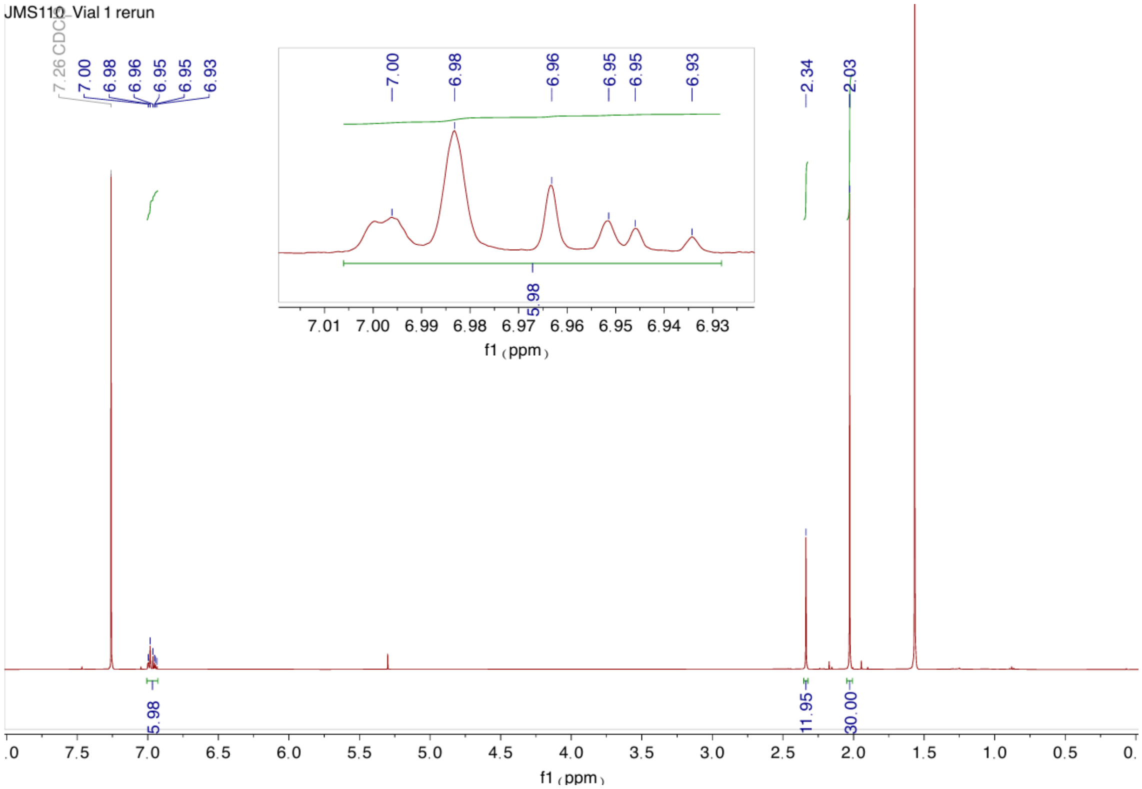

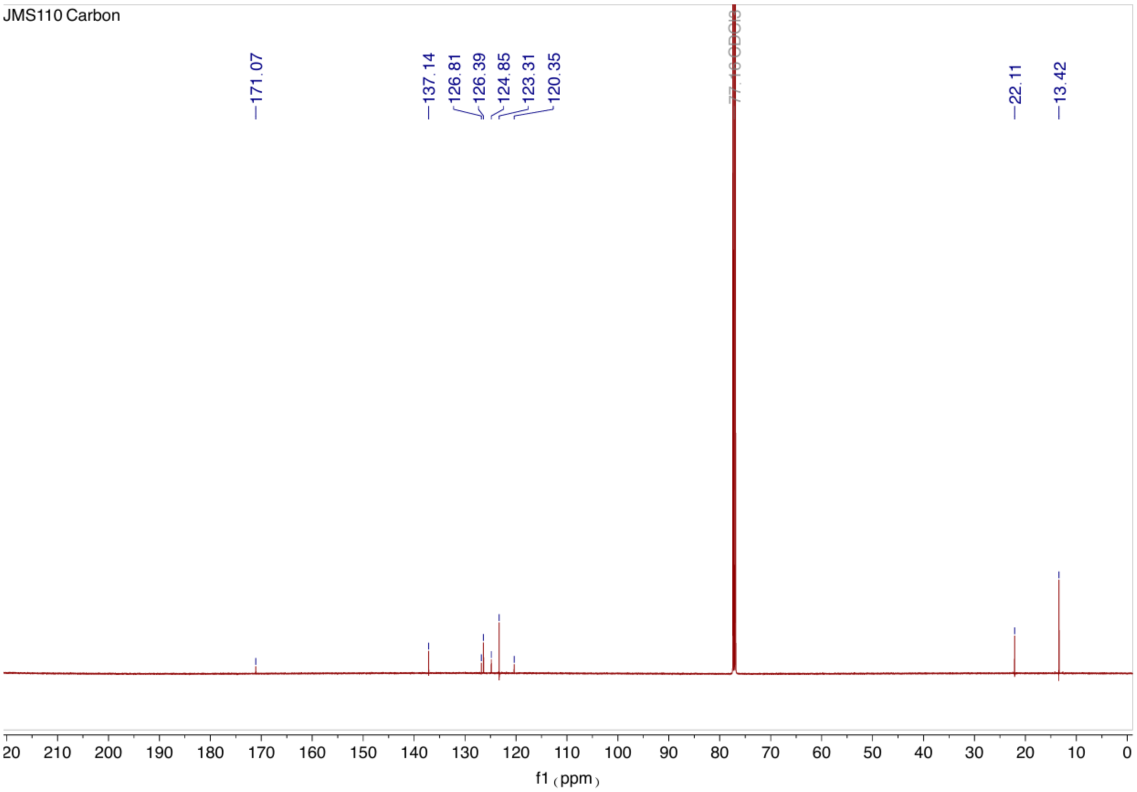

Figure S1. ^{1}H-NMR (500 MHz, $CDCl_3$, top) and ^{13}C-NMR (125 MHz, $CDCl_3$, bottom) of ^{xyl}**[Cp*Ti]**.

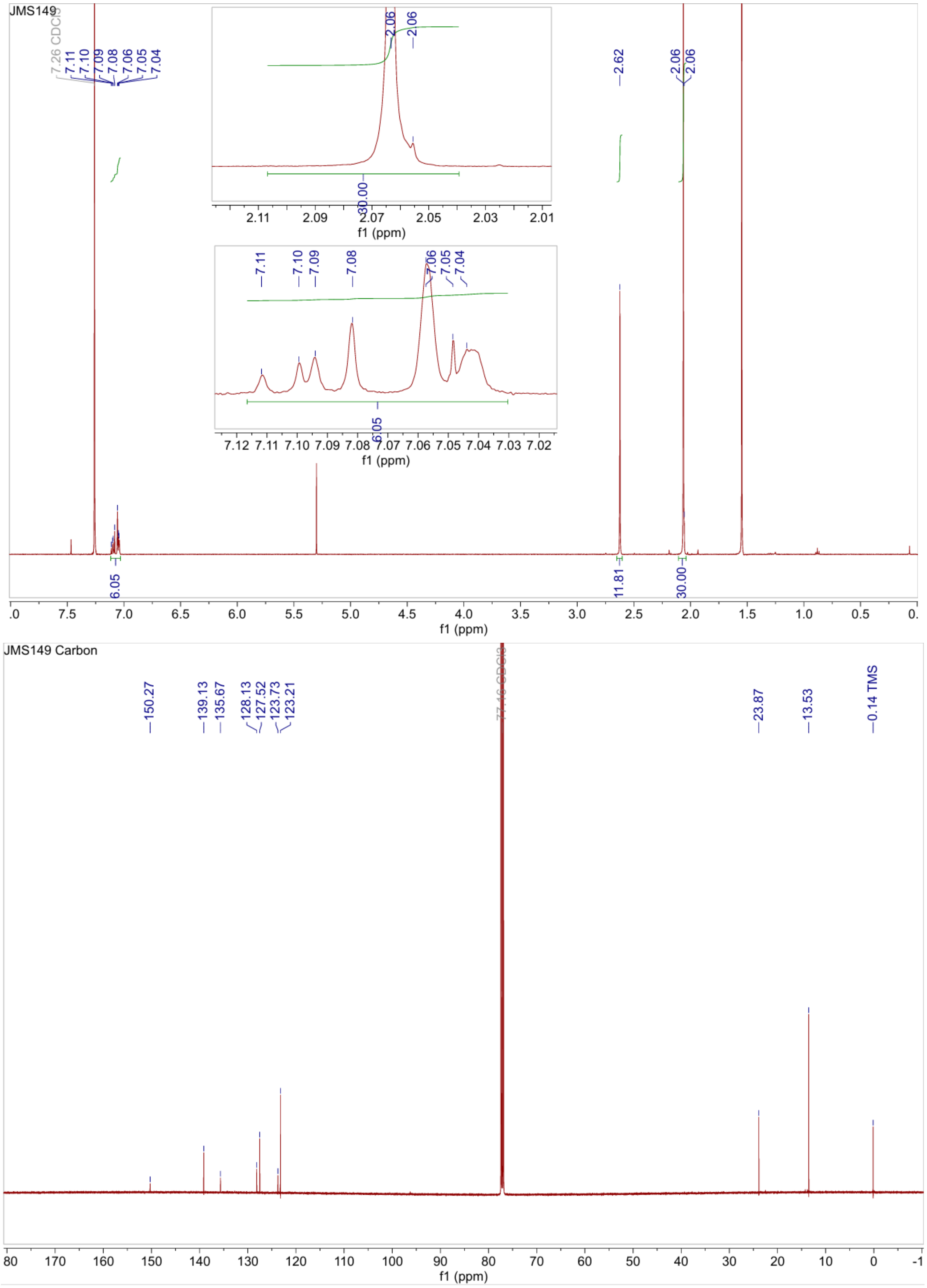

Figure S2. ^{1}H-NMR (500 MHz, $CDCl_3$, top) and ^{13}C-NMR (125 MHz, $CDCl_3$, bottom) of ^{xyl}**[Cp*Ti]CuBr.**

Table S1. Crystallographic data for **^{xyl}[Cp*Ti]CuBr**.

	**^{xyl}[Cp*Ti]CuBr**
empirical formula	$C_{40}H_{48}BrCuTi$
formula weight (g/mol)	720.13
crystal system	monoclinic
space group, Z	$P2_1/n$, 4
temperature (K)	130(2)
a (Å)	10.1109(4)
b (Å)	15.0056(6)
c (Å)	22.5981(9)
β (°)	91.1943(16)
volume (Å^3)	3427.8(2)
D_{calc} (g/cm^3)	1.395
crystal size (mm)	0.08 x 0.10 x 0.14
absorption coefficient (mm^{-1})	2.046
F(000)	1496
Tmax, Tmin	1.000, 0.912
Θ range for data	3.26-27.50
reflections collected	85676
data/restraints/parameters	7844/0/402
R(int)	0.0823
R1, wR2 [$I > 2\sigma(I)$]	0.0364, 0.0793
R1, wR2 (all data)	0.0533, 0.0878
goodness-of-fit on F^2	1.030
largest difference peak, hole (eÅ^{-3})	0.496, -0.347
CCDC Deposition No.	2440925

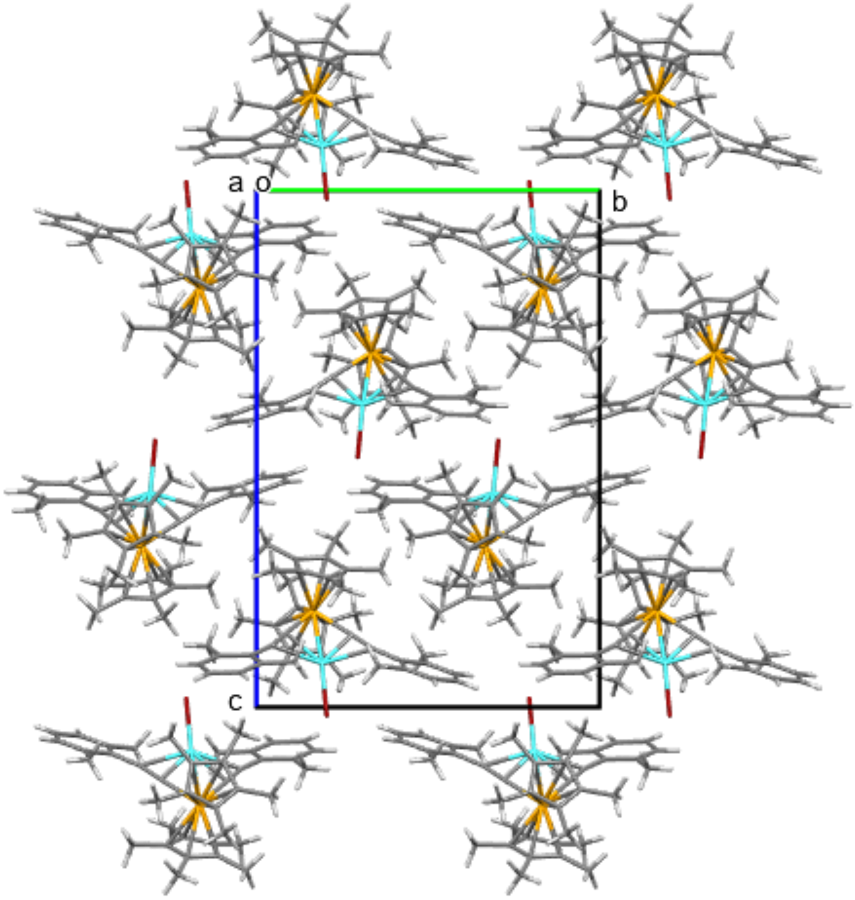

Figure S3. Packing of ^{xyl}**[Cp*Ti]CuBr** complexes viewed along the a-axis.

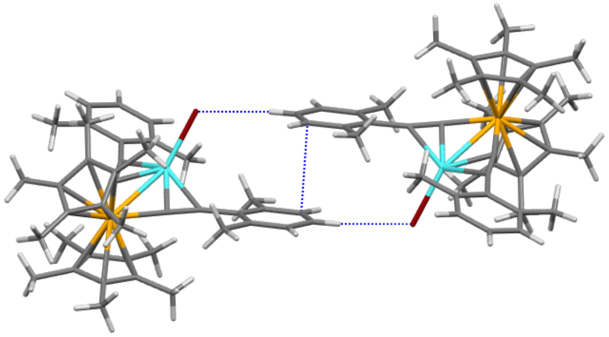

Figure S4. Dimer unit of ^{xyl}**[Cp*Ti]CuBr** with C-H⋯Br interactions and offset π-stacking noted with blue dashed lines.

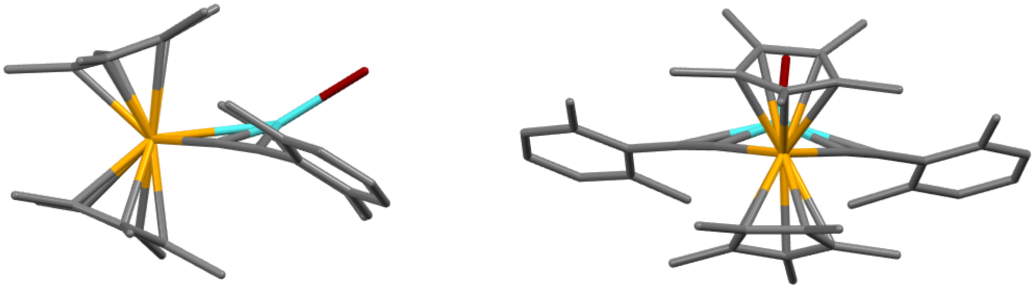

Figure S5. Tube diagrams showing different orientations for **^{xyl}[Cp*Ti]CuBr**. Cu deviates from the C-Ti-C plane by 0.50 Angstroms; Br deviates from the C-Ti-C plane by 1.85 Angstroms.

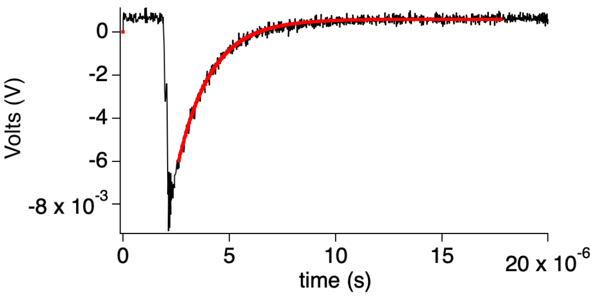

Figure S6. Luminescence decay trace for **^{xyl}[Cp*Ti]CuBr** in argon purged THF solution at RT (λ_{ex} = 367 nm, λ_{em} = 736 nm). A single exponential fit to the decay is shown in red. Data was collected using the OLIS SM-45 EM fluorescence lifetime system described in the Time-Resolved Emission section of the Experimental.

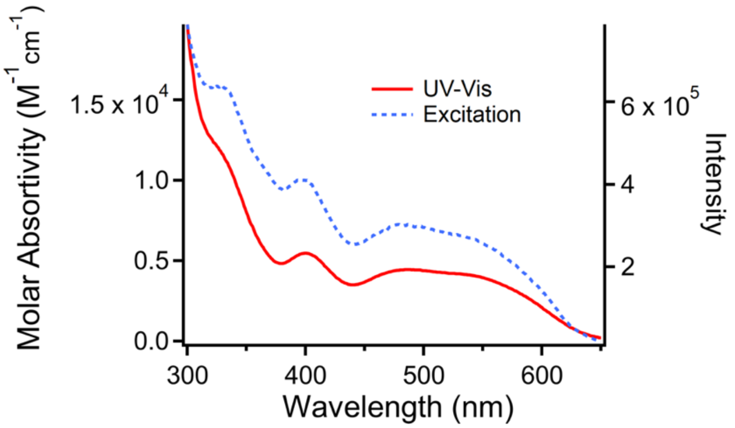

Figure S7. Absorption and excitation spectrum (λ_{em} = 736 nm) for **^{xyl}[Cp*Ti]CuBr** in THF solution at RT.

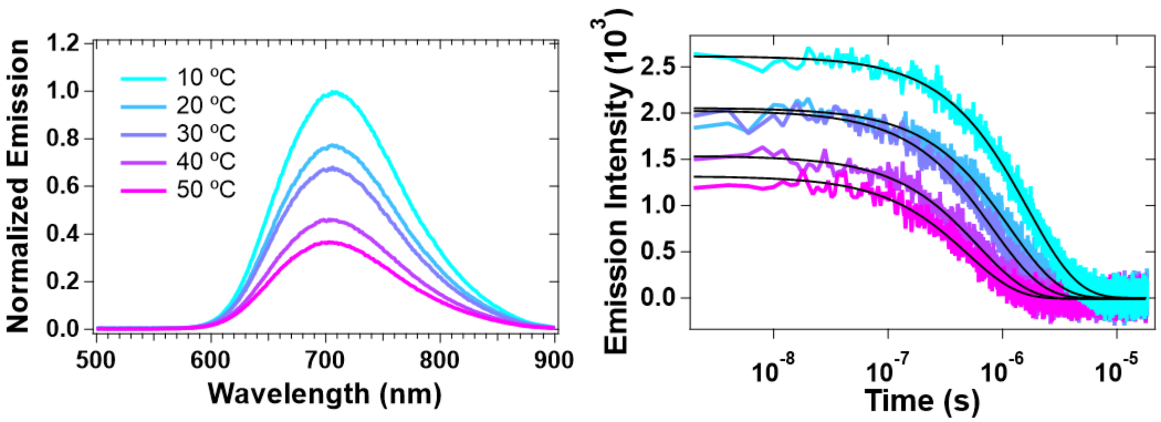

Figure S8. Emission spectra (left) and kinetics at 700 nm (right) of **^{xyl}[Cp*Ti]CuBr** in nitrogen deaerated THF as a function of temperature (λ_{ex} = 570 nm, A_{570} = 0.05). Single exponential fits to the decays are shown in black.

Table S2. Temperature dependent emission fit parameters used to derive the Arrhenius plot in **Figure S9**.[a]

Temperature (°C)	Temperature (K)	1/Temp (K^{-1})	τ (ns)	*k* (s^{-1})	ln(*k*)
10	283.15	0.003532	1670.2	598730.7	13.30257
20	293.15	0.003411	1160.1	861994.7	13.667
30	303.15	0.003299	825.7	1211094	14.00703
40	313.15	0.003193	617.6	1619171	14.29742
50	323.15	0.003095	492.8	2029221	14.52316

[a] Data collected using the Edinburgh Instruments LP980-KS spectrometer system described in the Temperature-Dependent Emission section of the Experimental.

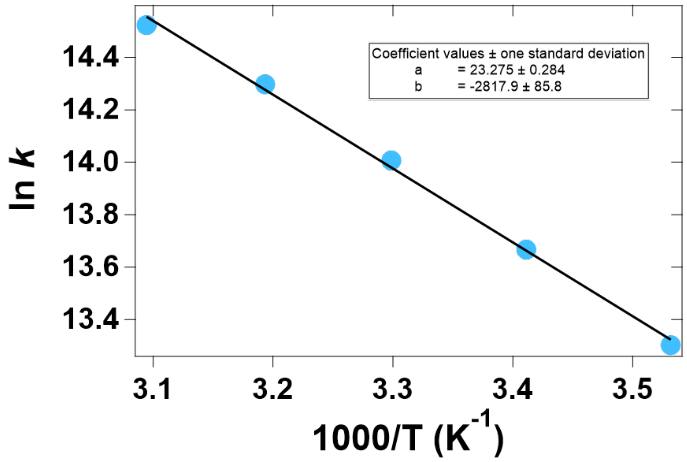

Figure S9. Arrhenius plot for temperature-dependent emission decay kinetics for **^{xyl}[Cp*Ti]CuBr** in nitrogen deaerated THF (λ_{em} = 700 nm).

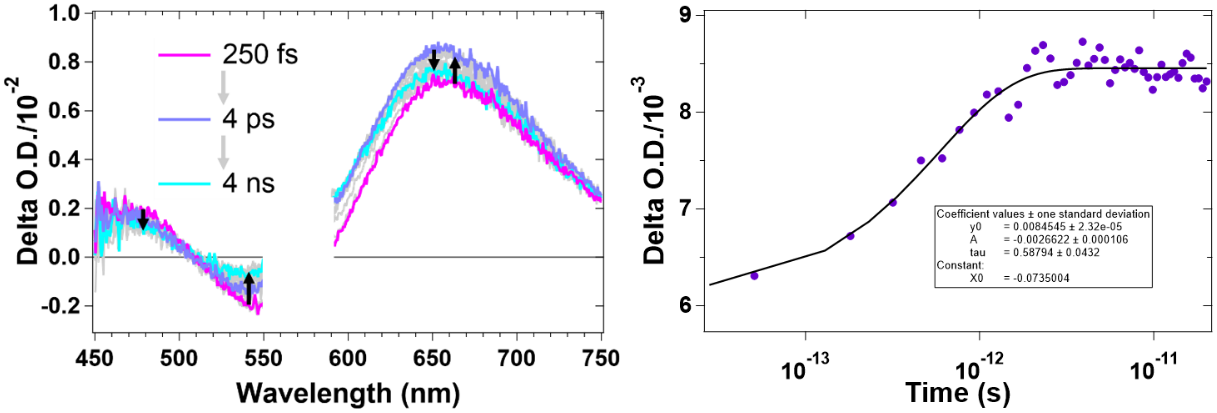

Figure S10. Left: transient absorption spectra for ^{xyl}**[Cp*Ti]CuBr** in THF (500 μM) at RT (λ_{ex} = 570 nm, A_{570} = 0.025). Right: single-wavelength kinetic and fit at 660 nm.

Table S3. Exponential decay lifetimes for the fits to the excited state absorptions and Figure 5.

Wavelength (nm)	τ (ns)
370	916 ± 13
450	909 ± 10
650	913 ± 5

Table S4. Key Interatomic Distances and Angles for ^{xyl}[Cp*Ti]CuBr.

Geometric Parameters	X-Ray	DFT
Ti-Cu	2.890	2.929
Ti-C_{Alkyne}	2.115, 2.105	2.132, 2.135
Ti-$Cp^*_{Centroid}$	2.105, 2.099	2.156, 2.151
Cu-Br	2.338	2.403
C≡C	1.223,1.228	1.239
C-C_{Cp^*}	1.416, 1.419	1.424, 1.425
Ti-Cu-Br ∠	154.62°	157.29°
Cp*-Ti-Cp* ∠	139.20°	137.79°
Ti-C≡C ∠	172.70°, 174.75°	174.95°, 173.82°

Chart S1. Orbital contributions to the singlet transitions and lowest-energy triplet transition for **^{xyl}[Cp*Ti]CuBr**

Excited State 1: Triplet-A 1.8785 eV 660.02 nm f=0.0000 <S**2>=2.000
187 -> 188 0.69184

Excited State 3: Singlet-A 2.1094 eV 587.77 nm f=0.0218 <S**2>=0.000
183 -> 188 -0.12828
187 -> 188 0.68331

Excited State 5: Singlet-A 2.2627 eV 547.94 nm f=0.0819 <S**2>=0.000
185 -> 188 0.16073
186 -> 188 0.66892

Excited State 8: Singlet-A 2.4164 eV 513.10 nm f=0.0030 <S**2>=0.000
179 -> 188 -0.11147
184 -> 188 -0.30566
185 -> 188 0.60010
186 -> 188 -0.11715

Excited State 9: Singlet-A 2.4861 eV 498.70 nm f=0.0265 <S**2>=0.000
184 -> 188 0.60974
185 -> 188 0.29509
186 -> 188 -0.14552

Excited State 11: Singlet-A 2.6281 eV 471.76 nm f=0.0193 <S**2>=0.000
181 -> 188 -0.13620
183 -> 188 0.66197
187 -> 188 0.12498

Excited State 13: Singlet-A 2.7897 eV 444.44 nm f=0.0022 <S**2>=0.000
181 -> 188 0.52896
182 -> 188 -0.42744
183 -> 188 0.11819

Excited State 16: Singlet-A 3.0097 eV 411.95 nm f=0.0180 <S**2>=0.000
176 -> 188 -0.29164
179 -> 188 0.51795
180 -> 188 -0.33097
185 -> 188 0.11288

Excited State 18: Singlet-A 3.0542 eV 405.95 nm f=0.0592 <S**2>=0.000
178 -> 188 0.10225
181 -> 188 0.41441
182 -> 188 0.53058

Excited State 22: Singlet-A 3.3428 eV 370.90 nm f=0.0059 <S**2>=0.000
175 -> 188 0.55784
177 -> 188 0.37058
187 -> 189 0.16042

Excited State 26: Singlet-A 3.4224 eV 362.27 nm f=0.0771 <S**2>=0.000
177 -> 188 -0.20583
187 -> 189 0.61823

Excited State 29: Singlet-A 3.4587 eV 358.47 nm f=0.0248 <S**2>=0.000
175 -> 188 -0.13150
176 -> 188 0.14568

177 -> 188 0.16360
178 -> 188 -0.13802
186 -> 189 0.59765

Excited State 31: Singlet-A 3.4713 eV 357.17 nm f=0.0163 <S**2>=0.000
175 -> 188 -0.32796
177 -> 188 0.38738
178 -> 188 -0.30307
186 -> 189 -0.24832
187 -> 189 0.16042

Excited State 34: Singlet-A 3.5417 eV 350.07 nm f=0.0019 <S**2>=0.000
174 -> 188 -0.13894
176 -> 188 0.26196
177 -> 188 0.12627
178 -> 188 -0.10825
179 -> 188 0.41463
180 -> 188 0.40544
187 -> 190 -0.17136

Excited State 35: Singlet-A 3.5435 eV 349.89 nm f=0.0047 <S**2>=0.000
176 -> 188 0.41220
177 -> 188 -0.10145
180 -> 188 -0.40835
187 -> 190 -0.29989

Excited State 37: Singlet-A 3.5734 eV 346.97 nm f=0.0194 <S**2>=0.000
175 -> 188 -0.16974
177 -> 188 0.20903
178 -> 188 0.48458
185 -> 189 -0.20257
186 -> 189 0.10246
187 -> 190 -0.28394

Excited State 38: Singlet-A 3.5804 eV 346.29 nm f=0.0404 <S**2>=0.000
175 -> 188 -0.11698
176 -> 188 0.21950
177 -> 188 0.16914
178 -> 188 0.32400
179 -> 188 0.10769
184 -> 189 0.19864
185 -> 189 0.32266
186 -> 189 -0.14881
187 -> 190 0.25617

Excited State 39: Singlet-A 3.6140 eV 343.06 nm f=0.1163 <S**2>=0.000
174 -> 188 0.19767
176 -> 188 -0.17753
184 -> 189 0.43465
184 -> 191 0.13572
185 -> 189 0.26769
186 -> 189 0.11140
187 -> 190 -0.29127
187 -> 192 -0.10188

Excited State 42: Singlet-A 3.6782 eV 337.08 nm f=0.0376 <S**2>=0.000
179 -> 189 -0.11600
184 -> 189 -0.42669

185 -> 189 0.44697
187 -> 190 -0.20214

Excited State 44: Singlet-A 3.7826 eV 327.77 nm f=0.0122 <S**2>=0.000
174 -> 188 0.62622
176 -> 188 0.21978

Excited State 46: Singlet-A 3.8404 eV 322.84 nm f=0.0522 <S**2>=0.000
181 -> 189 -0.13955
185 -> 190 -0.13056
186 -> 190 0.62499
186 -> 192 0.10111

Excited State 47: Singlet-A 3.8536 eV 321.74 nm f=0.0001 <S**2>=0.000
182 -> 189 0.20198
183 -> 189 0.51099
183 -> 191 0.12961
185 -> 190 -0.20331
187 -> 191 -0.20780
187 -> 194 -0.16832

Excited State 50: Singlet-A 3.9445 eV 314.32 nm f=0.0015 <S**2>=0.000
181 -> 189 0.47443
181 -> 191 0.10489
182 -> 189 -0.19223
183 -> 189 0.21570
184 -> 190 0.24583
185 -> 190 -0.12897
186 -> 190 0.11620
187 -> 194 0.17619

Excited State 52: Singlet-A 3.9559 eV 313.41 nm f=0.0091 <S**2>=0.000
183 -> 189 0.20778
183 -> 194 -0.13098
184 -> 190 -0.31417
184 -> 192 -0.13542
186 -> 190 -0.17165
187 -> 191 0.36850
187 -> 194 0.33558

Excited State 54: Singlet-A 4.0271 eV 307.87 nm f=0.0065 <S**2>=0.000
181 -> 190 -0.22945
181 -> 192 -0.12557
182 -> 190 0.12056
183 -> 190 0.11437
183 -> 193 -0.14428
187 -> 192 0.36309
187 -> 193 0.45623

Excited State 56: Singlet-A 4.0354 eV 307.24 nm f=0.0156 <S**2>=0.000
173 -> 188 0.66973
182 -> 188 0.10478

Excited State 58: Singlet-A 4.0745 eV 304.29 nm f=0.0207 <S**2>=0.000
181 -> 190 -0.10244
182 -> 190 0.19882
183 -> 190 0.51868
183 -> 192 0.14619

187 -> 190 0.18711
187 -> 192 -0.21862

Excited State 60: Singlet-A 4.1091 eV 301.73 nm f=0.1540 <S**2>=0.000
181 -> 189 0.16373
182 -> 189 0.22126
183 -> 189 0.14386
185 -> 190 0.53389

Chart S2. Orbital contributions to the singlet transitions and lowest-energy triplet transition for **Ph[Cp*Ti]CuBr**

Excited State 1: Triplet-A 2.1475 eV 577.35 nm f=0.0000 <S**2>=2.000
170 -> 172 -0.26443
171 -> 172 0.63267

Excited State 4: Singlet-A 2.3713 eV 522.86 nm f=0.0104 <S**2>=0.000
170 -> 172 0.62467
171 -> 172 -0.30747

Excited State 7: Singlet-A 2.5086 eV 494.23 nm f=0.1444 <S**2>=0.000
168 -> 172 -0.23740
170 -> 172 0.28783
171 -> 172 0.58642

Excited State 8: Singlet-A 2.5312 eV 489.83 nm f=0.0202 <S**2>=0.000
168 -> 172 0.55055
169 -> 172 -0.36322
170 -> 172 0.10378
171 -> 172 0.19720

Excited State 9: Singlet-A 2.5553 eV 485.20 nm f=0.0038 <S**2>=0.000
168 -> 172 0.35384
169 -> 172 0.58551

Excited State 12: Singlet-A 2.8566 eV 434.03 nm f=0.0057 <S**2>=0.000
165 -> 172 -0.41363
166 -> 172 0.55762

Excited State 13: Singlet-A 2.9582 eV 419.12 nm f=0.0013 <S**2>=0.000
158 -> 172 -0.13840
167 -> 172 0.68266

Excited State 18: Singlet-A 3.2222 eV 384.79 nm f=0.0039 <S**2>=0.000
162 -> 172 0.45627
163 -> 172 0.50578

Excited State 19: Singlet-A 3.2528 eV 381.16 nm f=0.1986 <S**2>=0.000
165 -> 172 0.54879
166 -> 172 0.40520

Excited State 20: Singlet-A 3.3358 eV 371.68 nm f=0.1376 <S**2>=0.000
164 -> 172 0.68564

Excited State 23: Singlet-A 3.4416 eV 360.25 nm f=0.0336 <S**2>=0.000
159 -> 172 -0.31958
162 -> 172 0.48845

163 -> 172 -0.37984

Excited State 29: Singlet-A 3.7644 eV 329.36 nm f=0.0012 <S**2>=0.000
168 -> 173 0.15623
169 -> 173 0.46793
170 -> 173 0.14024
171 -> 173 0.43562

Excited State 32: Singlet-A 3.8042 eV 325.91 nm f=0.0014 <S**2>=0.000
168 -> 173 0.48952
168 -> 174 0.10453
169 -> 173 -0.12110
170 -> 174 0.27288
170 -> 175 0.17373
170 -> 176 -0.23665
171 -> 174 -0.12066

Excited State 33: Singlet-A 3.8179 eV 324.75 nm f=0.0469 <S**2>=0.000
168 -> 174 0.12039
168 -> 175 0.12639
168 -> 176 -0.15115
169 -> 173 0.10463
170 -> 173 0.54440
171 -> 173 -0.25970

Excited State 36: Singlet-A 3.8962 eV 318.22 nm f=0.0076 <S**2>=0.000
158 -> 172 0.52007
159 -> 172 0.27497
160 -> 172 0.14561
161 -> 172 0.23513
167 -> 172 0.14511

Excited State 38: Singlet-A 3.9492 eV 313.95 nm f=0.0478 <S**2>=0.000
159 -> 172 -0.11251
168 -> 173 0.39708
170 -> 174 -0.31711
170 -> 175 -0.20112
170 -> 176 0.24116
171 -> 173 -0.11840
171 -> 174 0.14904
171 -> 176 -0.13461

Excited State 40: Singlet-A 3.9796 eV 311.55 nm f=0.0069 <S**2>=0.000
158 -> 172 -0.15491
159 -> 172 0.19691
163 -> 172 -0.10557
169 -> 173 -0.40014
170 -> 173 0.21781
171 -> 173 0.40652

Excited State 42: Singlet-A 4.0309 eV 307.58 nm f=0.0584 <S**2>=0.000
158 -> 172 -0.21419
159 -> 172 0.47243
161 -> 172 -0.10945
162 -> 172 0.16231
163 -> 172 -0.21890
169 -> 173 0.15634
171 -> 173 -0.19188

171 -> 174 -0.12346
171 -> 175 0.10925

Excited State 43: Singlet-A 4.0392 eV 306.95 nm f=0.0021 <S**2>=0.000
165 -> 173 -0.31545
166 -> 173 0.37128
169 -> 174 -0.21689
169 -> 175 -0.14173
169 -> 176 0.19151
170 -> 174 -0.11193
170 -> 176 0.12555
171 -> 174 -0.18318
171 -> 175 -0.14177
171 -> 176 0.11339

Excited State 45: Singlet-A 4.0802 eV 303.87 nm f=0.0008 <S**2>=0.000
160 -> 172 0.56699
161 -> 172 -0.40048

Excited State 47: Singlet-A 4.1014 eV 302.30 nm f=0.0088 <S**2>=0.000
158 -> 172 -0.31423
160 -> 172 0.35924
161 -> 172 0.48831

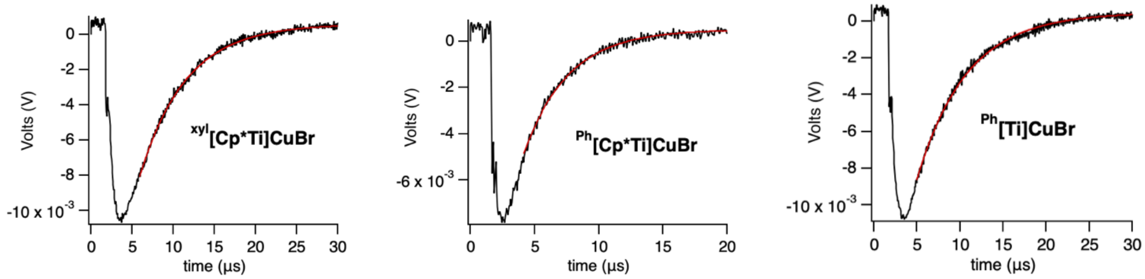

Figure S11. Luminescence decay traces (λ_{ex} = 367 nm) for **^{xyl}[Cp*Ti]CuBr**, **^{Ph}[Cp*Ti]CuBr**, and **^{Ph}[Ti]CuBr** in PMMA films (5 wt %) at RT, overlaid with a first-order exponential fit in red. Data collected and fit using the OLIS SM-45 EM fluorescence lifetime system described in the Time-Resolved Emission section of the Experimental.

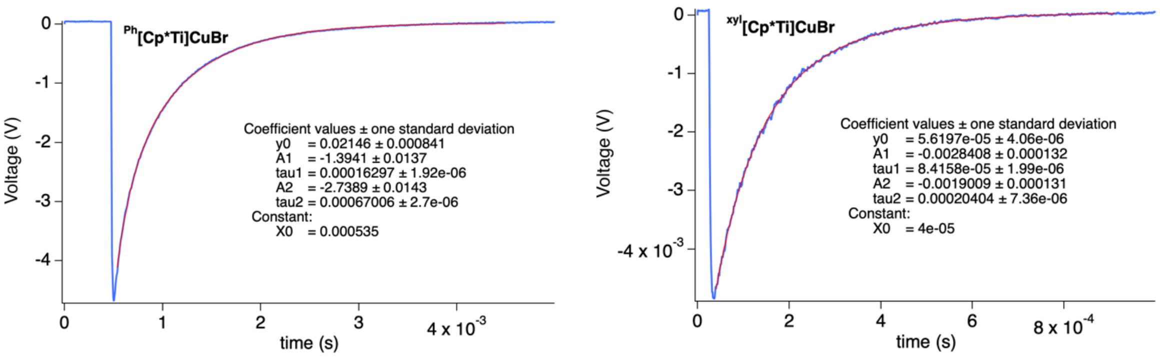

Figure S12. Luminescence decay traces (λ_{ex} = 367 nm) for **^{Ph}[Cp*Ti]CuBr** and **^{xyl}[Cp*Ti]CuBr** in 77 K matrix of 2-methyltetrahydrofuran overlaid with double exponential fits performed using IGOR pro. Data collected using the OLIS SM-45 EM fluorescence lifetime system described in the Time-Resolved Emission section of the Experimental.

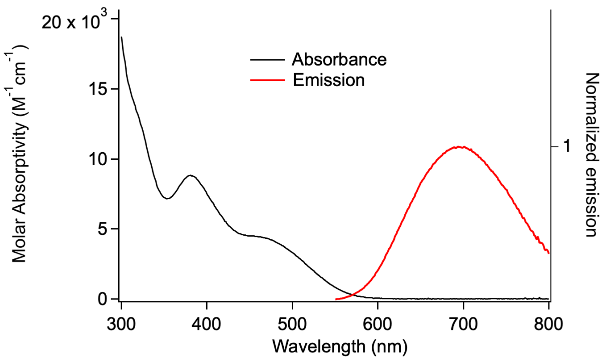

Figure S13. Absorption and emission spectra for **^{Ph}[Cp*Ti]CuBr** in THF solution at room temperature. From reference 53.

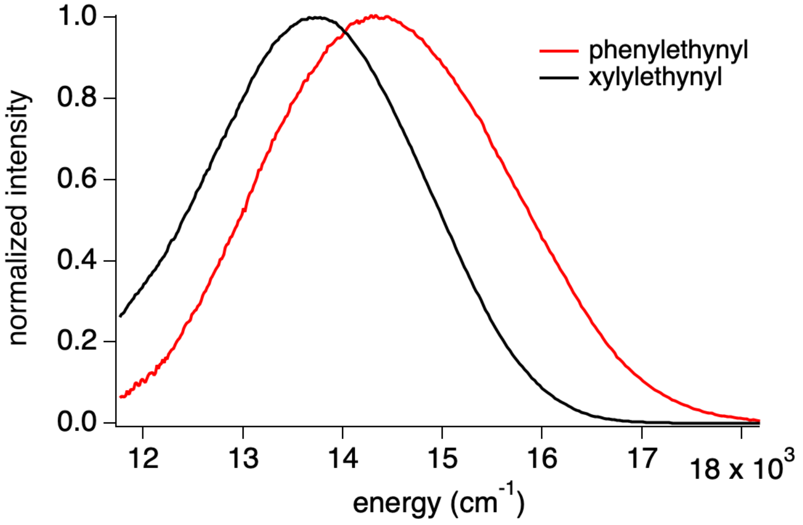

Figure S14. Emission spectra for **^{Ph}[Cp*Ti]CuBr** (red trace) and **^{xyl}[Cp*Ti]CuBr** (black trace) in THF solution at room temperature. Solutions were absorbance matched at the excitation wavelength (A_{380} = 0.23) and run with identical slit parameters (excitation slits = 3 nm, emission slits = 10 nm) using identical background subtraction and correction files. The x-axis was converted to units of wavenumbers after data collection.

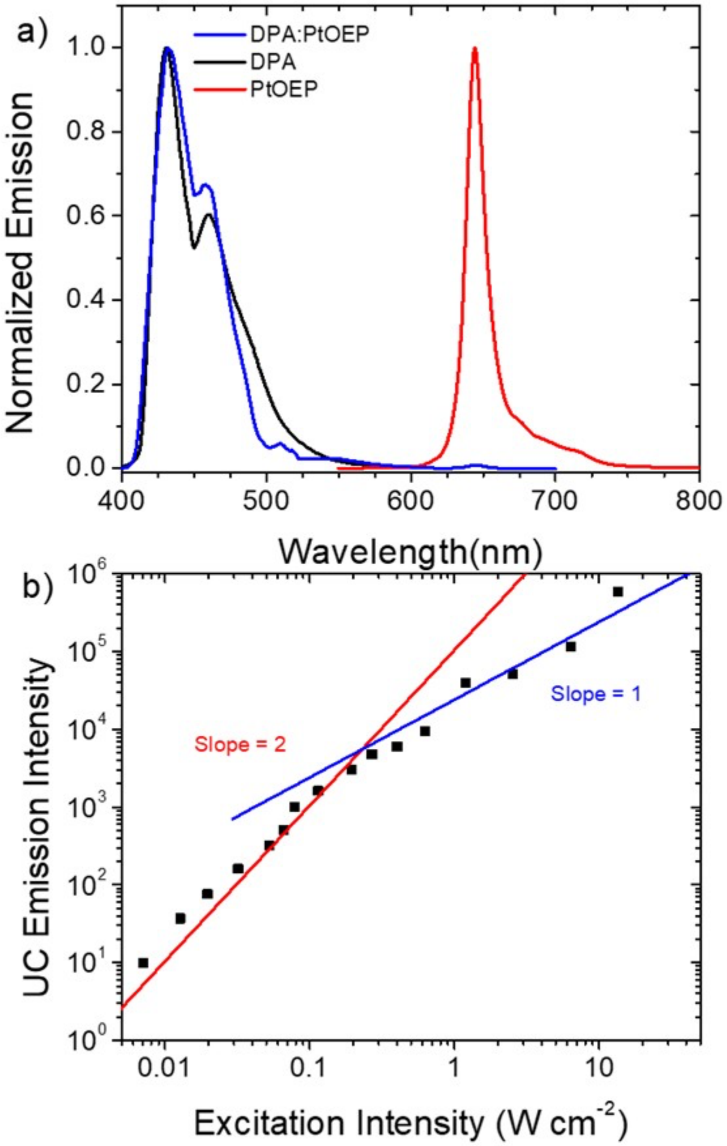

Figure S15. a) Normalized emission spectra for **DPA** (black, λ_{ex} = 350nm), **PtOEP** (red, λ_{ex} = 532 nm, 13.53 W cm^{-2}) and 10 mM:100 μM **DPA**:**PtOEP** (blue, λ_{ex} = 532 nm, 13.53 W cm^{-2}) in nitrogen deaerated THF. b) Emission intensity for the same solution at 430 nm (black square) with respect to 532 nm excitation intensity. Lines with a slope of 1 (blue) and 2 (red) are overlaid on the data.

Table S5. Lifetime of ^{xyl}**[Cp*Ti]CuBr** in THF at RT as a function of $[O_2]$.[a]

	$[O_2]$[b]	Lifetime (μs)	k_{obs}
Argon	0	1.55	6.46×10^5
Air	2.10×10^{-3}	1.33	7.55×10^5
O_2	1.00×10^{-2}	0.813	1.23×10^6

[a] Data collected using the OLIS SM-45 EM fluorescence lifetime system described in the Time-Resolved Emission section of the Experimental (λ_{ex} = 367 nm). [b] Oxygen concentrations in air and oxygen saturated THF solutions taken from Gonzalez-Carrero, S.; de la Guardia, M.; Galian, R. E.; Perez-Prieto, J. Pyrene-Capped CdeSe@ZnS Nanoparticles as Sensitive Flexible Oxygen Sensors in Non-Aqueous Media. *ChemistryOpen* **2014**, *3*, 199 – 205.

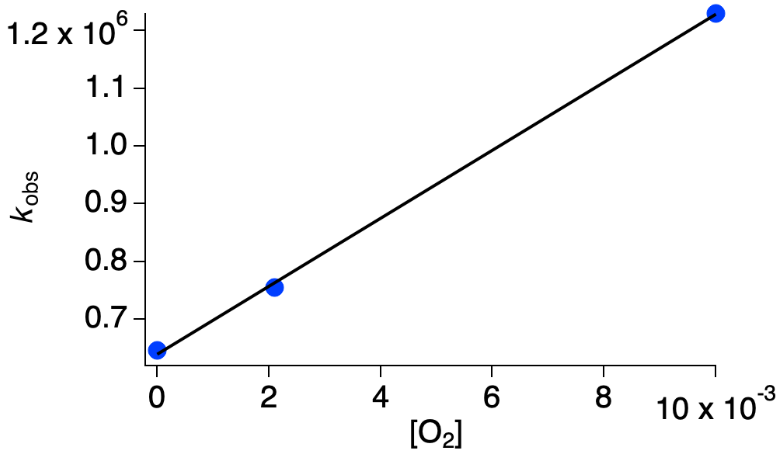

Figure S16. Stern-Volmer plot for quenching of ^{xyl}**[Cp*Ti]CuBr** by oxygen in THF solution at RT.
